# A national survey of anthelmintic resistance in ascarid and strongylid nematodes in Australian Thoroughbred horses

**DOI:** 10.1016/j.ijpddr.2023.11.006

**Published:** 2023-11-29

**Authors:** Ghazanfar Abbas, Abdul Ghafar, Emma McConnell, Anne Beasley, Jenni Bauquier, Edwina J.A. Wilkes, Charles El-Hage, Peter Carrigan, Lucy Cudmore, John Hurley, Charles G. Gauci, Ian Beveridge, Elysia Ling, Caroline Jacobson, Mark A. Stevenson, Martin K. Nielsen, Kristopher J. Hughes, Abdul Jabbar

**Affiliations:** a Melbourne Veterinary School, The University of Melbourne, Werribee, Victoria 3030, Australia; b Centre for Animal Production and Health, Murdoch University, Murdoch, Western Australia, Australia; c School of Agriculture and Food Sustainability, University of Queensland, Gatton, Queensland 4343, Australia; d Racing Victoria, Flemington, Victoria 3031, Australia; e Scone Equine Hospital, Scone, New South Wales 2337, Australia; f Swettenham Stud, Nagambie, Victoria 3608, Australia; g M.H. Gluck Equine Research Center, Department of Veterinary Science, University of Kentucky, Lexington, KY, USA; h School of Agricultural, Environmental and Veterinary Sciences, Charles Sturt University, Wagga Wagga, New South Wales 2650, Australia

**Keywords:** Horse, Cyathostomins, *Parascaris* spp., Anthelmintic resistance, Egg reappearance period, Macrocyclic lactones, Australia

## Abstract

This study quantified the extent of anthelmintic resistance (AR) in ascarid and strongylid nematodes against commonly used anthelmintics in Australian Thoroughbred horses. Faecal egg count reduction tests (FECRTs, *n* = 86) and egg reappearance period (ERP) tests were conducted on 22 farms across Australia. Faecal egg counts (FECs) were determined using the modified McMaster technique, and percent faecal egg count reduction (%FECR) was calculated using the Bayesian hierarchical model and hybrid Frequentist/Bayesian analysis method. The results were interpreted using old (published in 1992) and new (2023) research guidelines of the World Association for the Advancement of Veterinary Parasitology (WAAVP). The species composition of strongylid nematodes was detected utilising a DNA-metabarcoding method using pre- and post-treatment samples. Resistance was observed in strongylid nematodes to commonly used single-active and combination anthelmintics, including ivermectin (IVM %FECR range: 82%–92%; 95% lower credible interval (LCI) range: 80%–90%), abamectin (ABM: 73%–92%; 65%–88%), moxidectin (MOX: 89%–91%; 84%–89%), oxfendazole (OFZ: 0%–56%; 0%–31%) and its combination with pyrantel (OFZ + PYR: 0%–82%; 0%–78%). Resistance in *Parascaris* spp. was observed to IVM (10%–43%; 0%–36%), ABM (0%; 0%) and MOX (0%; 0%). When the new thresholds recommended by the WAAVP were used, AR was detected in six additional FECRTs for strongylids and three more tests for *Parascaris* spp., introducing resistance to OFZ and OFZ + PYR in the latter. Shortened ERPs (4–6 weeks) of strongylids were observed in 31 FECRTs in which AR was not detected at 2 weeks post-treatment for all the anthelmintics tested. Among cyathostomins, *Cylicocyclus nassatus*, *Cylicostephanus longibursatus* and *Coronocyclus coronatus* were the most prevalent species at 2 weeks post-treatment, whereas the main species appearing at five weeks following treatments with macrocyclic lactones were *Cylicocyclus nassatus, Cylicostephanus longibursatus* and *Cylicocyclus ashworthi*. After treatment with OFZ + PYR, the latter three, plus *Coronocyclus coronatus* and *Cyathostomum catinatum*, were detected at 5 weeks post-treatment. Overall, the study highlights the prevalence of AR in both ascarids and strongylid nematodes against commonly used anthelmintic products to control worms in Australian horses. The results indicate that ML combination products provided acceptable efficacy at 2 weeks. However, ERP calculations suggest that products work less effectively than previously measured. It is suggested to regularly monitor the efficacy of the anthelmintics and consider changing the worm control practices to better manage worms and AR in Australian horses.

## Graphical abstract

**Figure:**
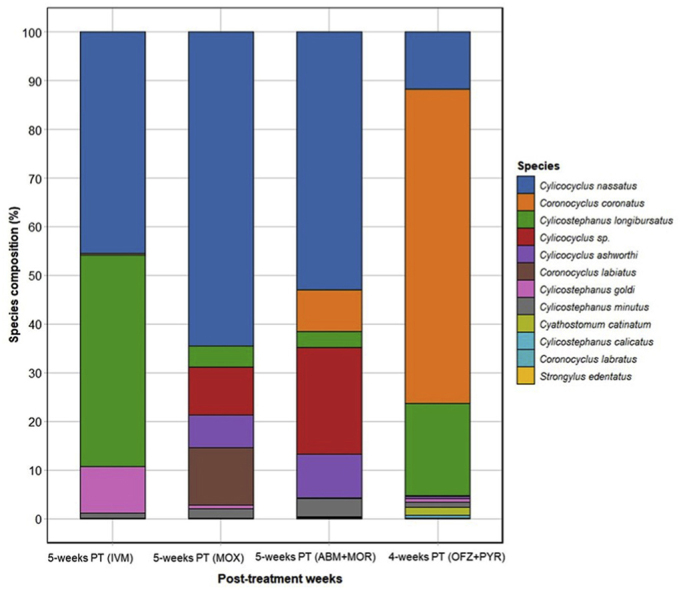

## 1 Introduction

Horses can be infected with various gastrointestinal nematodes (GINs), but cyathostomins (Strongylida: Cyathostominae) and *Parascaris* spp. (Ascaridida: Ascarididae) are the most common parasites of horses worldwide. Cyathostomins are ubiquitous parasites of all age groups of horses, and it is common for horses to be infected with 15–25 species (Bellaw and Nielsen, 2020) which can result in a disease syndrome, larval cyathostominosis, due to a synchronous emergence of cyathostomin larvae from the intestinal wall of horses (Reid et al., 1995; Love et al., 1999). Ascarids (*Parascaris equorum* and *P. univalens*) primarily infect foals and weanlings, and can lead to poor growth, colic and intestinal obstruction (Cribb et al., 2006). Horses develop protective, strong immunity against ascarids by one year of age, thereby significantly reducing the occurrence of patent infection in horses beyond two years of age (Reinemeyer, 2012).

Since the introduction of broad-spectrum anthelmintics in the 1960s, parasite control in horses has mainly been based on frequent, prophylactic interval-based deworming (Kaplan and Nielsen, 2010; Matthews, 2014). The adoption of other approaches for parasite control, such as husbandry and farm management, has been low among horse farm managers and owners (O'Meara and Mulcahy, 2002; Saeed et al., 2019; Wilkes et al., 2020). The frequent and indiscriminate use of anthelmintics has resulted in the development of anthelmintic resistance (AR) in ascarids and cyathostomins against the commonly used anthelmintics, including benzimidazoles (BZs), tetrahydropyrimidines (THPs) and macrocyclic lactones (MLs) (Nielsen, 2022; Macdonald et al., 2023). The patterns for the development of AR in ascarids and cyathostomins are of importance, as there is widespread resistance to MLs in *Parascaris* spp. and emerging resistance to BZs and THPs (Armstrong et al., 2014; Nielsen, 2016; Martin et al., 2018). Conversely, for cyathostomins, AR is well-established against BZs and THPs and resistance is emerging against MLs (Peregrine et al., 2014; Saeed et al., 2019; Nielsen, 2022). Recently, resistance in cyathostomins following treatments with moxidectin (MOX) has been increasingly reported (Nielsen et al., 2020; Flores et al., 2020; Abbas et al., 2021; Lignon et al., 2021; Martins et al., 2021). This situation is of considerable concern as MOX is often the only effective drug available against encysted larval cyathostomins in addition to 5-day fenbendazole (FBZ) treatment (Reinemeyer et al., 2015; Bellaw et al., 2018). Furthermore, no new anthelmintic class of drugs has been developed for use in horses during the last four decades, further increasing the concerns for sustainable worm control in the horse industry.

To circumvent the problem of AR in horse parasitic nematodes, alternative control strategies have been developed to slow the development and spread of AR. For example, evidence-based deworming, i.e., selective treatment, where only high egg shedders are dewormed to reduce the number of eggs/larvae on the pastures and associated infection pressures (Nielsen et al., 2014). However, the uptake of such programs is very low among horse owners and managers (O'Meara and Mulcahy, 2002; Wilkes et al., 2020), resulting in routine reliance on anthelmintics. Combinations of different anthelmintic drug classes have been used in horses in Australia and elsewhere to address the issue of increasing AR to single anthelmintic drugs. For instance, various combinations of MLs and THPs, and BZs and THPs are commonly used on Australian Thoroughbred farms (Beasley et al., 2017, 2020; Wilkes et al., 2017, 2020; Abbas et al., 2023a). The use of combinations of anthelmintics is widely recommended in the sheep industry to slow the development of AR and has contributed to successful parasite control in sheep (Le Jambre et al., 2010; Bartram et al., 2012). Some studies in horses have reported an additive effect when two drugs from different classes are used together. For example, an enhanced efficacy was observed when a combination of oxibendazole (from the BZ drug class) and pyrantel pamoate (from the THP drug class) was used against strongyles and *Parascaris* spp. in horses (Kaplan et al., 2014; Lyons et al., 2016). However, the drug combinations may not be sustainable when resistance has already developed to both anthelmintics (Scare et al., 2018). This also necessitates the regular monitoring of the efficacy of combinations of anthelmintics.

The measurement of the egg reappearance period (ERP) of strongylids following anthelmintic treatments has been considered an early indicator of the development of AR (Sangster, 1999). A recent study, however, has suggested that there may be other plausible reasons for a shortened ERP (Nielsen et al., 2022b), necessitating further research to understand the underlying mechanisms involved (Macdonald et al., 2023). Due to a lack of consensus on the definition of ERP, previous studies do not allow elucidation of the underlying reasons for the shortening of ERP in nematode populations (Macdonald et al., 2023). The recent guidelines for evaluating the efficacy of equine anthelmintics by the World Association for the Advancement of Veterinary Parasitology (WAAVP) provided a new definition of the ERP (Nielsen et al., 2022c) to facilitate meaningful comparisons between different studies and an understanding of the mechanism behind the shortening of ERPs. The WAAVP also recommends that drug efficacy test(s) should also investigate the species composition of strongylid nematodes before and after treatment to understand individual species' role in the development of AR (Nielsen et al., 2022c; Kaplan et al., 2023). To date, several molecular techniques have been used to identify GINs of horses to species level, including conventional and quantitative polymerase chain reaction, restriction fragment length polymorphism and reverse line blot assay. However, each technique has advantages and disadvantages for identifying parasites (Courtot et al., 2023; Ghafar et al., 2023). Recently, a high-throughput next-generation sequencing (NGS) tool, targeting the second internal transcribed spacer (ITS-2) of the nuclear ribosomal DNA and a partial mitochondrial cytochrome *c* oxidase subunit I (COI), has been developed to determine the species composition of nematodes in horse faecal samples and/or parasite material (Mitchell et al., 2019; Poissant et al., 2021; Courtot et al., 2023). While such approaches have been used in few initial studies (Johnson and Biddle, 2021; Nielsen et al., 2022b; Sargison et al., 2022; Abbas et al., 2023b), additional improvements have been suggested for both markers. However, the DNA pool extracted from strongylid eggs can be reliably used for metabarcoding and bioinformatics analyses (Courtot et al., 2023).

Although Australia has the second largest population of Thoroughbred horses globally (www.tbaus.com), no comprehensive study has been conducted to assess the efficacy of commonly used anthelmintics and identification of resistant species using advanced molecular methods. The previous studies in Australia were of regional scope, either focusing on one drug class or one type of parasite. By 2017, resistance in *Parascaris* spp. had been reported to FBZ, pyrantel (PYR), and ivermectin (IVM) in two studies (Armstrong et al., 2014; Wilkes et al., 2017), whereas resistance to oxibendazole (OBZ), morantel (MOR), and suspected resistance to IVM had been reported in cyathostomins (Pook et al., 2002; Edward and Hoffmann, 2008). One study reported a cyathostomin ERP of 6 and 12 weeks following treatments with IVM and MOX, respectively (Beasley et al., 2017) but this could be considered within a normal range (Nielsen et al., 2019).

Recently, we reported resistance in cyathostomins to abamectin (ABM), MOX and a combination of oxfendazole (OFZ) and PYR (OFZ + PYR) (Abbas et al., 2021). Based on these preliminary findings and the first report of resistance to ABM and MOX from Australia, we conducted a survey of AR in ascarids and cyathostomins in Australian Thoroughbred horses based on faecal egg count reduction tests (FECRT) in farmed horses. The specific aims of the study were to: (i) assess the efficacy of commonly used anthelmintics (i.e., based on recent worm control surveys (Abbas et al., 2023a)), including MLs, BZs and combinations of MLs + THPs, and BZs + THPs against cyathostomins and *Parascaris* spp.; (ii) measure ERP for cyathostomins following treatment with MLs, BZs and combinations of MLs + THPs, and BZs + THPs; and (iii) to characterise the species composition of strongylid nematodes using the ITS-2 based DNA metabarcoding technique before and after anthelmintic treatments in FECRT and ERP studies.

## 2 Methods

### 2.1 Selection of horse farms

Most of the Australian Thoroughbred breeding industry is located in regional areas. More than half of all breeding mares are owned by breeders having fewer than or equal to five horses. There are 664 stud farms located in Australia, second only to America in terms of Thoroughbred horse population of mares (*n* = 21,500) and stallions (*n* = 700) (https://www.tbaus.com; last access 24 October 2023). Most of the studs are located in New South Wales (*n* = 228), Victoria (*n* = 170), and Queensland (*n* = 113), and smaller numbers in Western Australia (*n* = 75), South Australia (*n* = 48), Tasmania (*n* = 29) and Australian Capital Territory (*n* = 1). The official Australian breeding season begins on 1 September each year and continues through to December while foals are born from 1 August. Horse parasites are managed by frequent, prophylactic interval-based deworming of all horses on farms (Wilkes et al., 2020; Abbas et al., 2023a).

**Fig. 1:**
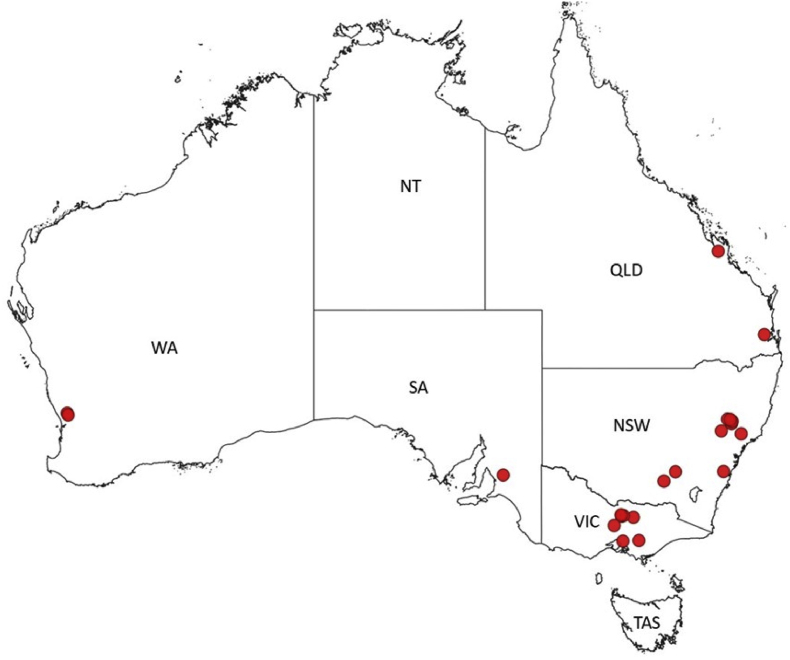
Map of Australia showing the locations of Thoroughbred horse farms (*n* = 22) enrolled for the efficacy of anthelmintics against ascarid and strongylid nematodes. Each red circle represents one Thoroughbred farm. Abbreviations: NSW, New South Wales; NT, Northern Territory; QLD, Queensland; VIC, Victoria; WA, Western Australia; SA, South Australia; TAS, Tasmania

Twenty-two Thoroughbred horse farms (designated as 1 to 22) were selected to take part in the FECRTs from June 2020 to December 2022 based on geographical locations across five states of Australia (Fig. 1). The farms were selected following longitudinal (Abbas et al., Unpublished) and cross-sectional (Abbas et al., 2023b) epidemiological studies of intestinal nematodes of Australian Thoroughbred horses where farm managers or resident veterinarians provided their consent to be contacted to participate in this study. Hard and soft copies of the study design, FECRT protocols, and a flyer showing dose adjustments for different dewormers were sent to the participating farms. The selection criteria used to enroll the farms in the study were: (i) the farm had at least 20 permanent resident horses at the time of study; (ii) horses had not been dewormed at least in the last 6 weeks; (iii) a confirmation that mean faecal egg counts (FEC) were greater than or equal to 30 eggs per gram (EPG) of faeces using the modified McMaster technique; and (iv) a known history of anthelmintic usage on the farms during the last five years. The collection of faecal samples from horses was approved by the Animal Ethics Committee of the University of Melbourne (Ethics ID: 1955146.1).

### 2.2 Faecal egg count reduction test and egg reappearance period

The FECRT was performed on each farm according to the WAAVP guidelines for detecting AR in nematodes of veterinary importance (Coles et al., 1992). To calculate the %FECR and ERP for strongylid nematodes, a total of 74 FECRTs were conducted at selected farms, including in foals (number of FECRTs = 2), weanlings (29), yearlings (40), and mares (3). Eight anthelmintic products (five single and three combinations) were used (Table 1), with four products (IVM, IVM + PYR, ABM, MOX) available in combination with praziquantel (PZQ). Given PZQ has no nematocidal activity, we have designated these combinations as IVM, IVM + PYR, ABM and MOX throughout the paper. Similarly, we conducted a total of 12 FECRTs to determine %FECR against *Parascaris* spp. for IVM (number of FECRTs = 3), ABM (1), MOX (1), OFZ (1), FBZ (1), OFZ + PYR (4) and ABM + MOR (1).

**Table 1:** Anthelmintics used in faecal egg count reduction tests conducted on Thoroughbred horse farms in Australia.

No. of farm(s) included	No. of test(s) conducted *(N*^a^*)*	Active drug(s) in anthelmintic products used	Brand name(s) of anthelmintic products used	Dose/kg body weight
12	15 (152)	Ivermectin	Equimec Paste^14^ Merial, Australia	0.2 mg
			Prazivec^1^, Riverside Veterinary, Australia	0.2 mg
3	4 (32)	Abamectin	Equimax^3^, Virbac Australia	0.2 mg
			MecWorma & Bot^1^, International Animal Health Products, Australia	0.2 mg
12	14 (141)	Moxidectin	Equest® Plus Tape, Zoetis Australia	0.4 mg
5	7 (46)	Oxfendazole	AMMO Rotational Wormer®, Ceva Animal Health Pty Ltd, Australia	10 mg
14	18 (169)	Oxfendazole and pyrantel embonate	Strategy-T®, Virbac Australia	10 mg oxfendazole + 6.6 mg pyrantel base
10	13 (142)	Abamectin and morantel tartrate	AMMO Allwormer Wormer^12^, Ceva Animal Health Pty Ltd, Australia	54 mg
			MecWorma & Tape^1^, International Animal Health Products, Australia	54 mg
2	2 (18)	Ivermectin, pyrantel embonate and praziquantel	Equimax Elevation, Virbac Australia	0.2 mg ivermectin +13 mg pyrantel
1	1 (12)	Fenbendazole	Panacur, Merck Animal Health, the USA	10 mg/kg for 5 days

Each superscript on brand name(s) of anthelmintic products indicates the number of trials conducted for that specific product when two different brands of the same active were used.

a Total number of horses included in the tests for an anthelmintic product.

Before the start of the study, FECs were performed on the selected farms to ascertain the faecal egg shedding in the horses selected for the study. Horses selected for the study were assigned to various treatment or control groups (where applicable) using simple randomisation. For *Parascaris* spp., the foals or weanlings were blocked according to age and then randomly selected for treatment and control groups (where possible). The control groups selected in FECRTs were to observe any natural variation in FECs of untreated horses during the study period. All horses included in the control groups were positive for faecal egg shedding and were treated at the end of the study. Anthelmintic dosage was calculated based on the individual horse body weight or weight of the heaviest animal within a treatment group using an electronic scale (where available at the farm) or a weight tape. In the majority of the tests, anthelmintics were administered by farm staff workers following the manufacturers' recommendations in the presence of the first author (G.A.) or by an equine veterinarian. The horses were given an additional dose of 10% to avoid underdosing.

The administration of anthelmintics and the collection of freshly voided faecal samples (or directly from the rectum of the horses where possible) were performed by the farm workers in the presence of a member of the research project team or by the first author in the majority of FECRTs. Fecal samples were collected on the day of anthelmintic treatment and 2 weeks post-treatment in all tests or up to 9 weeks post-treatment where the study was extended to observe the ERPs of cyathostomins. For this purpose, samples were collected on a weekly or biweekly basis, depending upon the availability of farm staff.

#### 2.2.1 Faecal egg counts

FECs were carried out within 48–96 h of the collection of faeces using the modified McMaster technique (Gordon and Whitlock, 1939; Ghafar et al., 2021). Briefly, four grams of faeces were mixed with four ml water to make a homogeneous slurry which was then mixed with 52 ml of sucrose solution (specific gravity 1.27, www.csrsugar.com.au) and homogenised using a spatula. Following homogenisation, a sample (1 ml volume) was pipetted into two chambers of a Whitlock egg counting slide (www.whitlock.com.au). After 10 min, eggs were counted using a compound light microscope. A multiplication factor of 15 for this method was applied to calculate the EPG.

#### 2.2.2 Interpretation of the FECRT and ERP results

The percentage faecal egg count reduction (%FECR) was calculated in all 86 tests to assess the efficacy of anthelmintics tested at two weeks post-treatment. Of the 74 FECRTs conducted for strongylids, 31 were extended up to 9 weeks post-treatment to determine cyathostomin ERPs. Group-based %FECRs and 95% credible intervals were calculated using the Bayesian hierarchical model in an online web interface (Torgerson et al., 2014; Wang et al., 2018) to ascertain the efficacy of an anthelmintic for each group. To interpret the efficacy results, a FECR threshold of >95% for MLs/combination drugs and >90% for BZs/THPs was used. Additionally, 95% lower credible intervals (LCIs) of 90 and 80 were selected for classifying resistance to MLs/combination drugs and BZs/THPs, respectively, as per the guidelines of the WAAVP (Coles et al., 1992). Resistance to a particular anthelmintic was confirmed if both thresholds were not achieved, while resistance was suspected when only one of the two criteria was met.

For the interpretation of ERP results, we adopted the standard definition of ERP as the time elapsed from day 0 to when %FECR returns to below 90% (von Samson-Himmelstjerna et al., 2007). In addition, we also used the latest ERP definition provided by WAAVP (i.e., when 95% UCL < %FECR at 2 weeks post-treatment – 10) to interpret ERP results (Nielsen et al., 2022c).

#### 2.2.3 Efficacy assessment of anthelmintics using new WAAVP guidelines

In addition to the above methods and thresholds used for calculating and classifying resistance or susceptibility profiling, we also compared old and new (research) thresholds provided in the recent WAAVP guidelines (Kaplan et al., 2023). This method involves the use of 90% CI instead of 95% CI to classify the efficacy outcomes. In a paired study design, the mean FEC data of pre- and post-treatment (EPG) were used to generate the efficacy profile through open-source software (available at https://www.fecrt.com) that uses the Bayesian-Frequentist Hybrid Inference method (Denwood et al., 2023). The research protocol was employed while selecting the drug class against cyathostomins and/or *Parascaris* spp. from drop-down list in the online interface and the efficacy classification was generated as resistant, low resistant, susceptible or inconclusive (Kaplan et al., 2023). The efficacy thresholds based on research protocol for various drug classes were as follows:

• For MLs against strongylids: a target (expected) efficacy of 99.9% and a lower efficacy threshold of 96%;
• For BZs against strongylids: a target (expected) efficacy of 99% and a lower efficacy threshold of 95%.
• For combination products: the thresholds of the single active within the combination with higher expected efficacy were used (e.g., if a combination of MLs and PYR was used, we applied the thresholds for MLs).
• For all drugs used against *Parascaris* spp.: a target (expected) efficacy of 99.9% and a lower efficacy threshold of 95%.

Given the latest WAAVP guidelines were not available at the time of the study, the study design may not be totally compliant. To check the statistical power of the test and extent of study compliance with new recommendations, the total number of eggs counted from pre-treatment samples in each group were compared with the group size. In addition, we calculated the required sample size for FECRTs (research protocol) using retrospective pre-treatment FEC data of individual tests via the open-source software available at https://www.fecrt.com (Denwood et al., 2023).

### 2.3 Identification of strongylid nematodes

#### 2.3.1 DNA extraction

We used high-throughput next-generation sequencing of the ITS-2 to determine the species composition of strongylid nematodes in horse faecal samples collected pre- and post-treatment. To reduce the number of DNA samples for testing using the PCR-directed NGS, we pooled the faecal samples after determining individual FECs based on pre- and post-treatment weeks per group for each FECRT. Following FECs of individual samples, 1–2 ml of supernatant from the faecal slurry was collected from each sample and pooled by the treatment group in a 50 ml Falcon tube for each batch of samples per drug test to extract the eggs as previously described by Roeber et al. (2013), with some modifications. The Falcon tube was then filled with floatation solution to make a total volume of 50 ml. Following centrifugation at 760×*g* for 10 min, ∼1 ml of supernatant was collected into an Eppendorf microcentrifuge tube and stored at −20 °C for further use.

Following thawing, 250 μl of concentrated eggs were used to extract genomic DNA using the DNeasy PowerSoil extraction kit (Qiagen, Hilden, Germany) according to the manufacturer's protocol. The extracted DNA was stored at −20 °C for further processing. Based on the FECRT and ERP results, DNA was extracted from 2 to 5 weeks post-treatment samples. In addition, genomic DNA was also extracted from 20 samples available from a previous study (Abbas et al., 2021) to ascertain the species composition of cyathostomins resistant to ABM, ABM + MOR, MOX, OFZ and OFZ + PYR.

#### 2.3.2 PCR-based next-generation sequencing

The ITS-2 rDNA was amplified from genomic DNA extracted from pooled samples (total = 210: pre-treatment samples = 74; post-treatment samples = 136). A few samples (*n* = 26) were removed from the data during downstream workflow due to low or no amplification of DNA. Primary PCR amplification was achieved using the primers NC1 and NC2 (Gasser et al., 1993) with additional 5′-adaptor sequences (F: 5′-GTGACCTATGAACTCAGGAGTC-primer-3′; R: 5′-CTGAGACTTGCACATCGC-AGC-primer-3′). Each PCR reaction was carried out in a volume of 25 μl containing 3.12 mM each of deoxynucleotide triphosphate (dNTP), 6.25 pmol of each adaptor primer, 125 mM MgCl_2_, 5X GoTaq reaction buffer, and 0.6 U of GoTaq flexi DNA polymerase (Promega, Madison, WI, USA). The thermocycling conditions consisted of an initial denaturation for 5 min at 95 °C followed by 24 cycles of 95 °C (30 s), 55 °C (30 s) and 72 °C (30 s) followed by final elongation for 5 min at 72 °C. Three positive controls including single species of *Strongylus equinus*, *Coronocyclus* (*Cor.*) *coronatus* and a mixture of five species *Cylicocyclus* (*Cylc*.) *nassatus* and *Cylicostephanus* (*Cyls*.) *calicatus, Oesophagodontus robustus, Parapoteriostomum mettami and S. equinus*, and one (for PCR 1) and two (for PCR 2) negative controls (Ambion nuclease-free water- Life Technologies, Austin, TX, USA) were included in the NGS experiments. Amplicons were purified using 1X Ampure Beads (Beckman Coulter, Brea, CA, USA).

Secondary PCR was carried out to introduce 8-base forward and reverse indices (Illumina, San Diego, CA, USA) into individual amplicons (i.e., multiplexing) for subsequent sequencing of all amplicons in two runs. Forty-eight forward and 22 reverse indices were used, allowing the multiplexing of 205 amplicons (samples: 184; positive controls: 12; negative controls: 9). The confirmed positive and negative control samples used in the adaptor PCR as well as those of indexing PCR were used to exclude cross-contamination. Aliquots (10 μL) of each purified amplicon from the first PCR were used as a template along with 10 μL of OneTaq® 2X Master Mix (New England Biolabs, Ipswich, MA, USA) and 0.5 μL of each index. The thermocycling conditions were an initial denaturation for 3 min at 95 °C followed by 18 cycles of 95 °C (15 s), 60 °C (30 s) and 72 °C (30 s), and a final extension for 7 min at 72 °C.

Following an assessment of the quality of amplicons using an Agilent 2200 TapeStation (Agilent Technologies, Santa Clara, CA, USA), amplicons were purified using 1X Ampure Beads (Beckman Coulter, Brea, CA, USA). The aliquots (5 μL) of the purified amplicons were then pooled and sequenced using two 600-cycle (2 × 300 bp paired-end reads) kits (MiSeq Reagent Kit v3, Illumina San Diego, CA, USA) on a MiSeq platform (Illumina, San Diego, CA, USA).

#### 2.3.3 Nemabiome analysis and taxonomic assignment

The Illumina MiSeq automatically separated all sequences by samples during post-run processing using recognised indices. This resulted in FASTQ files comprising raw overlapping paired-end reads. All protocols were carried out following Illumina's standard MiSeq operating protocol. The paired-end FASTQ files were uploaded into QIIME2 v. 2021.11 environment (Bolyen et al., 2019) for downstream analyses. Subsequently, a summary of the overall reads was generated to visualise the distribution of sequence quality at each position. The adaptors and primers were trimmed from all forward and reverse reads using the cutadapt plugin in QIIME2 (Martin, 2011). Trimmed reads were then imported as QIIME2 compatible artifacts (.qza file extension). Quality plots were generated for randomly selected 10,000 sequences each from forward and reverse reads separately to assess the read quality in q2view (https://view.qiime2.org). The resulting interactive quality plots allowed the selection of truncation parameters to remove reads of low quality. Considering the length of our expected amplicons (between 250 and 410 bp), we selected both forward and reverse reads of above 35 quality score to proceed with the best quality reads.

The DADA2 plugin (Callahan et al., 2016) was used to remove chimeras and low-quality reads and to dereplicate and merge paired-end reads which resulted in amplicon sequence variants (ASVs), allowing a comparative measure of sequence diversity (Callahan et al., 2017). The nematode ITS-2 reference database (Workentine et al., 2020) was downloaded from the website (https://www.nemabiome.ca; last accessed on March 14, 2022). This version of the database was updated and used in the recent horse nemabiome studies (Poissant et al., 2021; Nielsen et al., 2022b) that contained 39 of the 50 species from Strongylidae (including *Cyathostomum montgomeryi*) known to infect horses globally. Therefore, the latest sequence library was used to classify the ASVs into species/taxa through the scikit-learn classifier (Pedregosa et al., 2011). An additional filtration step was applied in which only sequences classified as belonging to the families Strongylidae and Chabertiidae were selected. Subsets of ASVs were selected for each species/taxon and taxonomic assignments were verified using BLASTn (NCBI). A taxonomic bar plot of ASVs was generated using the QIIME2 taxa bar plot. Finally, a biological observation matrix (BIOM) table was generated that describes the presence or absence and/or abundance of different taxa across multiple samples (Dhariwal et al., 2017). Sample metadata information was added to the BIOM table for further downstream analysis in ‘R software’ version 4.2.1 (R Core Team 2023). The taxonomy data associated with the nemabiome community was also exported along with BIOM table.

Nemabiome data were further analysed using the contributed phyloseq package (McMurdie and Holmes, 2013) in R. To normalise the sample counts, each count (x) was divided by the sum of all counts in the pooled sample to represent the relative abundance of each taxon in the sample. To avoid the skewed distribution of certain taxa, data were transformed by taking the square root of each count. Stacked bar plots of the relative abundance of each taxon in each treatment group were generated using grouped data by pre-treatment, and 2 and 5 weeks post-treatment.

## 3 Results

### 3.1 Anthelmintic efficacy against strongylids

A total of 74 FECRTs were conducted to assess the efficacy of eight anthelmintic drugs against cyathostomins across five different states of Australia, including New South Wales (*n*umber of tests = 35), Victoria (*n* = 27), Queensland (*n* = 7), Western Australia (*n* = 4), and South Australia (*n* = 1). Anthelmintic resistance was detected in 33 of the 74 FECRTs (45%, 95% CI 34%–56%), with the highest number of tests positive for AR recorded in New South Wales (17 of 35, 49%, 95% CI 33%–64%) and Victoria (12 of 27, 44%, 95% CI 28%–63%) (Table 2).

**Table 2:** Outcome of faecal egg count reduction tests conducted to assess the efficacy of various anthelmintics against strongylid nematodes at Thoroughbred farms across Australia.

Active drug(s) of anthelmintics used	Total number of tests *(N*^a^*)*	State-wise distribution of tests with resistance or suspected resistance					% tests with resistance or suspected resistance (proportion)
		Victoria	New South Wales	Queensland	Western Australia	South Australia	
Ivermectin	15 (152)	1	2	0	0	0	20 (3/15)
Abamectin	4 (32)	1	2	0	0	0	75 (3/4)
Moxidectin	14 (141)	2	2	0	0	0	29 (4/14)
Oxfendazole	7 (46)	3	2	2		0	100 (7/7)
Fenbendazole	1 (12)	0	1	0	0	0	100 (1/1)
Oxfendazole and pyrantel embonate	18 (169)	5	8	1	1	0	83 (15/18)
Abamectin and morantel tartrate	13 (142)	0	0	0	0	0	0 (0/13)
Ivermectin, pyrantel embonate and praziquantel	2 (18)	0	0	0	0	0	0 (0/2)
**Total**	**74**	**12**	**17**	**3**	**1**	**0**	**45 (33/74)**

a Total number of horses included in the tests for an anthelmintic product.

#### 3.1.1 MLs single actives and ML-containing combinations

A total of 33 FECRTs were performed to assess the efficacy of IVM (*n* = 15), ABM (*n* = 4), and MOX (*n* = 14) against strongylid nematodes (Table 3). Ivermectin-resistant parasites were detected on two farms in weanlings and yearlings in New South Wales while resistance was suspected on one farm in yearlings in Victoria (Table 3). Similarly, ABM resistance was observed on two farms: weanlings in Victoria and mares in New South Wales while resistance was suspected in yearlings on the latter farm (Farm 21) (Table 3). Resistance to MOX was observed on three properties in weanlings in New South Wales, and weanlings and yearlings in Victoria. Fifteen FECRTs conducted to assess the efficacy of combinations products, i.e., ABM + MOR (13) and IVM + PYR (2) were found effective at 2 weeks post-treatment, with %FECR estimates between 97% and 100% (Table 4).

**Table 3:** Efficacy of treatments with macrocyclic lactones against strongylid nematodes in Thoroughbred horses across Australia.

Drugs used and farm codes	State	Age group	No. of animals included in each test	Mean faecal egg counts (eggs per gram)		% FECR (95% credible intervals)	Test outcome
				Pre-treatment	2 weeks post-treatment		
Ivermectin							
1	NSW	W	10	1717	303	82 (80–85)	**Resistant**
3	NSW	Y	6	1232	2	100 (99–100)	Susceptible
6	QLD	Y	7	802	15	98 (97–99)	Susceptible
8	WA	Y	7	877	10	99 (98–100)	Susceptible
9	VIC	M	9	818	1	100 (99–100)	Susceptible
10	VIC	M	13	985	4	100 (99–100)	Susceptible
11	VIC	Y	10	1515	86	94 (93–96)	**Suspected resistance**
13	NSW	W	7	1703	6	100 (99–100)	Susceptible
13	NSW	Y	7	989	71	93 (90–95)	**Resistant**
14	NSW	Y	15	1535	23	99 (98–99)	Susceptible
15	NSW	Y	12	1204	4.6	100 (99–100)	Susceptible
16	QLD	Y	9	1133	1	100 (100–100)	Susceptible
17	VIC	W	13	1,93	1	100 (98–100)	Susceptible
18	VIC	Y	12	155	1	100 (98–100)	Susceptible
18	VIC	W	15	1305	1	100 (99–100)	Susceptible
Abamectin							
12^a^	VIC	W	5	810	220	73 (65–79)	**Resistant**
21	NSW	Y	12	8,87	69	92 (90–94)	**Suspected resistance**
21	NSW	M	9	526	42	92 (88–95)	**Resistant**
22	SA	Y	6	1407	63	96 (94–97)	Susceptible
Moxidectin							
4	NSW	Y	10	1507	109	93 (91–94)	**Suspected resistance**
5	NSW	W	13	802	69	91 (89–94)	**Resistant**
6	QLD	Y	7	1721	8	100 (99–100)	Susceptible
8	WA	Y	7	1768	8	100 (99–100)	Susceptible
12^a^	VIC	W	5	511	3	100 (98–100)	Susceptible
12^a^	VIC	Y	5	579	2	100 (99–100)	Susceptible
13	NSW	Y	7	758	11	99 (97–100)	Susceptible
14	NSW	Y	15	969	4	100 (99–100)	Susceptible
15	NSW	Y	15	1114	2	100 (100–100)	Susceptible
18	VIC	Y	14	643	1	100 (100–100)	Susceptible
18	VIC	W	15	1004	4	100 (100–100)	Susceptible
19	NSW	Y	16	1065	5	100 (100–100)	Susceptible
20^a^	VIC	W	5	1053	120	89 (85–92)	**Resistant**
20^a^	VIC	Y	7	1477	143	90 (88–93)	**Resistant**

NSW, New South Wales; QLD, Queensland; SA, South Australia; VIC, Victoria; WA, Western Australia; %FECR, percent faecal egg count reduction; Y, yearlings; W, weanlings; M, mares; the outcome of FECRT was declared as susceptible, resistant, or suspected resistance based on Coles et al. (1992).

a Samples included from the previous study (Abbas et al., 2021).

**Table 4:** Efficacy of treatments with combinations of macrocyclic lactones and tetrahydropyridines against strongylid nematodes in Thoroughbred horses across Australia.

Drugs used and farm codes	State	Age group	No. of animals included in each test	Mean faecal egg counts (eggs per gram)		% FECR (95% credible intervals)	Test outcome
				Pre-treatment	2 weeks post-treatment		
Abamectin and morantel tartrate							
1	NSW	Y	12	1189	2.1	100 (99–100)	Susceptible
2	NSW	Y	21	2114	1	100 (100–100)	Susceptible
4	NSW	Y	10	1616	32	98 (97–99)	Susceptible
5	NSW	W	14	827	1	100 (100–100)	Susceptible
6	QLD	W	6	373	13	97 (93–99)	Susceptible
12^a^	VIC	W	5	201	3	100 (94–100)	Susceptible
12^a^	VIC	W	5	616	2	100 (98–100)	Susceptible
13	NSW	W	8	1996	1	100 (100–100)	Susceptible
13	NSW	Y	5	1187	2	100 (99–100)	Susceptible
15	NSW	Y	15	1475	33	98 (97–99)	Susceptible
17	VIC	W	19	886	1	100 (100–100)	Susceptible
19	NSW	Y	15	1498	1	100 (100–100)	Susceptible
20	VIC	F	7	2342	1	100 (100–100)	Susceptible
Ivermectin and pyrantel embonate							
4	NSW	Y	10	1608	1	100 (100–100)	Susceptible
13	NSW	W	8	1210	1	100 (100–100)	Susceptible

NSW, New South Wales; QLD, Queensland; VIC, Victoria; %FECR, percent faecal egg count reduction; Y, yearlings; W, weanlings; F, foals; the outcome of FECRT was declared as susceptible, resistant, or suspected resistance based on Coles et al. (1992).

a Samples included from the previous study (Abbas et al., 2021).

#### 3.1.2 BZs single actives and BZ-containing combinations

Eight FECRTs were conducted to assess the efficacy of OFZ (*n* = 7) and FBZ (*n* = 1) against strongylid nematodes and resistance to both drugs was observed in weanlings and yearlings on farms across News South Wales (*n* = 3), Queensland (*n* = 2) and Victoria (*n* = 3) (Table 5). Eighteen FECRTs were conducted to assess the efficacy of OFZ + PYR, and resistance was observed in foals, weanlings and yearlings in 15 tests across New South Wales (*n* = 8), Queensland (*n* = 1), Victoria (*n* = 5) and Western Australia (*n* = 1). However, three FECRTs revealed that OFZ + PYR was effective in foals, weanlings and yearlings in New South Wales, Victoria and Western Australia, respectively (Table 5).

**Table 5:** Efficacy of treatments with benzimidazole and their combinations with tetrahydropyridines against strongylid nematodes in Thoroughbred horses across Australia.

Drug(s) and farm code	State	Age group	No. of animals included in each test	Mean faecal egg counts (eggs per gram)		% FECR (95% credible intervals)	Test outcome
				Pre-treatment	2 weeks post-treatment		
Oxfendazole							
5	NSW	W	14	959	887	7 (0–14)	**Resistant**
6	QLD	W	5	461	394	14 (0–28)	**Resistant**
6	QLD	Y	6	1596	987	38 (31–46)	**Resistant**
12	VIC	W	5	69	33	56 (13–81)	**Resistant**
12^a^	VIC	W	5	990	974	0 (0–5)	**Resistant**
13	NSW	Y	6	103	473	0 (0–4)	**Resistant**
20	VIC	W	5	890	841	5 (0–15)	**Resistant**
Fenbendazole							
15	NSW	Y	12	1340	717	47 (41–52)	**Resistant**
Oxfendazole and pyrantel embonate							
1	NSW	Y	12	2128	1229	42 (37–47)	**Resistant**
2	NSW	W	11	1929	536	72 (69–75)	**Resistant**
3	NSW	F	7	427	2	100 (98–100)	Susceptible
4	NSW	Y	8	1869	503	73 (69–76)	**Resistant**
5	NSW	W	14	1751	1531	12 (5–18)	**Resistant**
6	QLD	Y	9	1206	984	19 (9–27)	**Resistant**
7	WA	Y	12	1493	1222	18 (11–25)	**Resistant**
8	WA	Y	6	322	16	95 (90–98)	Susceptible
9	VIC	W	12	1818	592	68 (64–71)	**Resistant**
10	VIC	Y	18	1505	304	80 (77–82)	**Resistant**
11	VIC	Y	11	1271	242	81 (78–84)	**Resistant**
12^a^	VIC	W	5	901	169	82 (76–86)	**Resistant**
12	VIC	W	5	491	26	95 (91–98)	Susceptible
13	NSW	Y	7	834	1228	0 (0–3)	**Resistant**
13	NSW	W	8	330	709	0 (0–3)	**Resistant**
14	NSW	W	10	2203	1231	44 (39–49)	**Resistant**
14	NSW	Y	7	1171	582	50 (43–57)	**Resistant**
20	VIC	W	7	301	294	8 (0–7)	**Resistant**

NSW, New South Wales; QLD, Queensland; VIC, Victoria; WA, Western Australia; %FECR, percent faecal egg count reduction; Y, yearlings; W, weanlings; F, foals; the outcome of FECRT was declared as susceptible, resistant, or suspected resistance based on Coles et al. (1992).

a Samples included from the previous study (Abbas et al., 2021).

### 3.2 Anthelmintic efficacy against Parascaris spp.

Twelve FECRTs were conducted to assess the efficacy of IVM (*n* = 3), ABM (*n* = 1), MOX (*n* = 1), OFZ (*n* = 1), FBZ (*n* = 1), ABM and MOR (*n* = 1), and OFZ + PYR (*n* = 4) against *Parascaris* spp. on nine farms across New South Wales (*n*o. of farms = 3) and Victoria (*n* = 6) (Table 6). Resistance in *Parascaris* spp. was only observed against IVM, ABM and MOX (Table 6). None of the horses in the control groups showed signs of a clinical disease associated with ascarids.

**Table 6:** Efficacy of single and combinations of anthelmintic drugs against ascarid nematodes in Thoroughbred horses across Australia.

Drug(s) and farm code	State	Age group	No. of animals included in each test	Mean faecal egg counts (eggs per gram)		% FECR (95% credible intervals)	*Test outcome*
				Pre-treatment	2 weeks post-treatment		
Ivermectin							
13	NSW	W	6	553	493	10 (0–23)	**Resistant**
17	VIC	W	13	663	25	96 (94–98)	Susceptible
18	VIC	F	8	1599	928	43 (36–48)	**Resistant**
Abamectin							
21	NSW	Y	6	521	511	0 (0–5)	**Resistant**
Moxidectin							
20	VIC	F	5	237	648	0 (0–0.4)	**Resistant**
Oxfendazole							
12	VIC	F	5	463	28	94 (89–97)	Susceptible
Fenbendazole							
18	VIC	F	6	1106	2	100 (99–100)	Susceptible
Oxfendazole and pyrantel embonate							
3	NSW	F	7	1825	21	99 (98–99)	Susceptible
9	VIC	W	12	286	1	100 (98–100)	Susceptible
10	VIC	W	9	597	14	98 (96–99)	Susceptible
12	VIC	F	5	939	39	96 (93–98)	Susceptible
Abamectin and morantel tartrate							
12	VIC	F	5	1367	2	100 (99–100)	Susceptible

NSW, New South Wales; VIC, Victoria; %FECR, percent faecal egg count reduction; Y, yearlings; W, weanlings; F, foals; the outcome of FECRT was declared as susceptible, resistant, or suspected resistance based on Coles et al. (1992).

### 3.3 Classification of anthelmintic efficacy using old and new (research) WAAVP thresholds

Of the 74 FECRTs conducted for strongylids, 30 and 36 tests resulted in resistant classification according to the old guidelines (Coles et al., 1992) and the new guidelines (Kaplan et al., 2023), respectively. Furthermore, using the new thresholds (Kaplan et al., 2023), outcomes of five tests were classified as low resistance (a new category introduced when the criteria for both resistance and susceptibility are met simultaneously), and four tests were deemed inconclusive (Table 7).

**Table 7:** Summary of drug efficacy outcomes based on the old and new (research) guidelines of the World Association for the Advancement of Veterinary Parasitology for faecal egg count reduction tests for strongylid and ascarid nematodes.

Drug	FECRTs conducted	Old method (Coles et al., 1992)			New method recommended for research (Kaplan et al., 2023)			
		Resistant	Suspected resistant	Susceptible	Resistant	Low resistant	Susceptible	Inconclusive
Strongylids								
IVM	15	2	1	12	3	4	7	1
ABM	4	2	1	1	4	0	0	0
MOX	14	3	1	10	4	1	9	0
ABM + MOR	13	0	0	13	1	0	10	2
IVM + PYR	2	0	0	2	0	0	2	0
OFZ	7	7	0	0	7	0	0	0
FBZ	1	1	0	0	1	0	0	0
OFZ + PYR	18	15	0	3	16	0	1	1
*Parascaris* spp.								
IVM	3	2	0	1	3	0	0	0
ABM	1	1	0	0	1	0	0	0
MOX	1	1	0	0	1	0	0	0
OFZ	1	0	0	1	1	0	0	0
FBZ	1	0	0	1	0	0	1	0
OFZ + PYR	4	0	0	4	1	0	2	1
ABM + MOR	1	0	0	1	0	0	1	0

FECRTs, faecal egg count reduction test; IVM, ivermectin; ABM, abamectin; MOX, moxidectin; MOR, morantel tartrate; PYR, pyrantel embonate; OFZ, oxfendazole; FBZ, fenbendazole.

Similarly, for the 12 FECRTs conducted for *Parascaris* spp., four and seven tests revealed resistance according to the old (Coles et al., 1992) and new guidelines (Kaplan et al., 2023), respectively. A drug-wise summary of these results is presented in Table 7, whereas detailed information on individual test results and comparisons of the old and new WAAVP guidelines can be found in Supplementary Tables S1–S4.

Calculations of required sample size corresponding to sufficient statistical power (≥80%) and mean number of eggs counted showed that most of the test groups were in compliance with the new recommendations of the WAAVP (Denwood et al., 2023; Kaplan et al., 2023) (see Supplementary Tables S1–S4).

### 3.4 Strongylid egg reappearance periods

Thirty-one FECRTs were continued to determine ERPs for IVM (*n* = 9), MOX (*n* = 9), ABM + MOR (*n* = 8), IVM + PYR (*n* = 2), and OFZ and PYR (*n* = 3) against strongylid nematodes (Table 8, Table 9). For IVM, the ERPs ranged from 4 to 6 weeks, with 4 and 5 weeks observed on three farms each, six weeks on one farm and no outcome was obtained on three farms due to discontinuation of sampling. There was a concordance in ERPs for IVM using both ERP calculation methods except in yearlings on one farm (Farm 13) in New South Wales where the ERP was four weeks using method 1 (i.e., when FECR <90%) and five weeks using method 2 (i.e., when 95% UCL < %FECR at 2 weeks post-treatment – 10) (Table 8). Similarly, the ERPs for MOX ranged from 4 to 6 weeks, with 4 and 5 weeks observed on two farms each, 6 weeks on three (using method 1) or four (using method 2) farms and no outcome was obtained on three farms due to discontinuation of sampling. Moxidectin remained effective beyond 9 weeks post-treatment on one farm (Farm 8) in Western Australia where this anthelmintic had never been used (Table 8).

**Table 8:** Egg reappearance periods (ERPs) of ivermectin and moxidectin against strongylid nematodes of Thoroughbred horses across Australia.

Drug and farm code	State	Age group	No. of animals included in each test	Frequency of sample collection	ERP calculation method 1^a^	ERP calculation method 2^b^
Ivermectin						
3	NSW	Y	6	Fortnightly	6 weeks	6 weeks
8	WA	Y	7	Weekly	5 weeks	5 weeks

13	NSW	Y	7	Weekly	4 weeks	5 weeks
13	NSW	W	7	Weekly	5 weeks	5 weeks

16	QLD	Y	9	Fortnightly	4 weeks	4 weeks
17	VIC	W	13	Fortnightly	4 weeks	4 weeks
18	VIC	Y	12	Weekly	5 weeks	5 weeks
Moxidectin						
4	NSW	Y	10	Fortnightly	4 weeks	4 weeks
6	QLD	Y	7	Fortnightly	6 weeks	6 weeks
8	WA	Y	7	Weekly	effective above 9 weeks	effective above 9 weeks
12^c^	VIC	W	5	Weekly	5 weeks	6 weeks
12^c^	VIC	Y	5	Weekly	5 weeks	NO
13	NSW	Y	7	Weekly	4 weeks	NO

18	VIC	Y	14	Weekly	6 weeks	6 weeks
18	VIC	W	15	Fortnightly	6 weeks	6 weeks

NO, no outcome due to discontinuation of samples; NSW, New South Wales; QLD, Queensland; VIC, Victoria; WA, Western Australia; Y, yearlings; W, weanlings; F, foals.

a When percent faecal egg count reduction (%FECR) < 90.

b When Upper 95% credible limit < %FECR at 2 weeks post-treatment – 10.

c Samples included from the previous study (Abbas et al., 2021).

**Table 9:** Egg reappearance periods (ERPs) of various combinations of anthelmintics against strongylid nematodes of Thoroughbred horses across Australia.

Drug and farm code	State	Age group	No. of animals included in each test	Frequency of sample collection	ERP calculation method 1^a^	ERP calculation method 2^b^
Abamectin and morantel tartrate						
4	NSW	Y	10	Fortnightly	4 weeks	NO
12^c^	VIC	W	5	Weekly	4 weeks	4 weeks
12^c^	VIC	W	5	Weekly	5 weeks	5 weeks
13	NSW	W	8	Weekly	5 weeks	5 weeks
15	NSW	Y	13	Fortnightly	4 weeks	NO
17	VIC	W	18	Fortnightly	6 weeks	6 weeks
20	VIC	F	7	Weekly	5 weeks	5 weeks
Oxfendazole and pyrantel embonate						
3	NSW	F	7	Weekly	6 weeks	6 weeks
8	WA	Y	6	Weekly	3 weeks	4 weeks
12	VIC	W	5	Weekly	4 weeks	5 weeks
Ivermectin and pyrantel embonate						
4	NSW	Y	10	Fortnightly	4 weeks	4 weeks
13	NSW	W	8	Fortnightly	6 weeks	6 weeks

NO, no outcome due to discontinuation of samples; NSW, New South Wales; VIC, Victoria; WA, Western Australia; Y, yearlings; W, weanlings; F, foals.

a When percent faecal egg count reduction (%FECR) < 90.

b When Upper 95% credible limit < %FECR at 2 weeks post-treatment – 10.

c Samples included from the previous study (Abbas et al., 2021).

The ERPs were also determined for combinations of anthelmintic drugs, with 4–6 weeks for ABM + MOR, 3–6 weeks for OFZ + PYR, and 4–6 weeks for IVM + PYR using method 1 (Table 9). Similar ERPs were calculated for these combinations using method 2 except in tests following ABM + MOR treatments where ERPs were inconclusive using method 2 due to the discontinuation of sampling after 4 weeks (Table 9). Similarly, ERPs of 4 weeks (Farm 8) and 5 weeks (Farm 12) were calculated in two tests following OFZ + PYR treatments which were calculated at 3 and 4 weeks using method 1 (Table 9). The tests were removed from Table 8, Table 9 where no outcome could be calculated due to the discontinuation of samples. These include tests for IVM at farms 11 and 15, MOX at farm 15, ABM + MOR at farm 2.

### 3.5 Nematode composition following treatments with MLs and their combinations with THPs

**Fig. 2:**
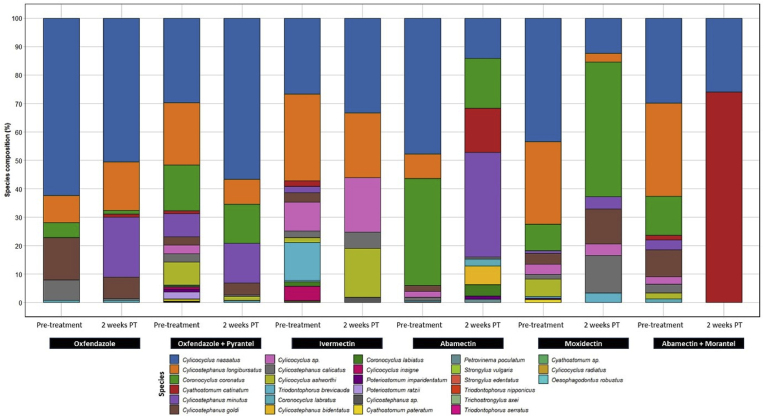
Relative abundance of strongylid nematodes in pooled faecal samples collected from pre- and post-treatment groups of horses following treatment with various anthelmintics. Each taxon is defined by one colour. In each bar, the mean nemabiome of each taxon is based on the total number of variants of one species divided by the total number of counts of all species.

The testing of 15 pre-treatment faecal DNA samples from IVM groups revealed 15 strongylid nematode species (Fig. 2), with the predominant species being *Cyls. longibursatus* (30.6%) *Cylc. nassatus* (26.6%), *Triodontophorus* (*T.*) *brevicauda* (13.5%), *Cyclicocyclus* sp. (10.1%), *Cyls. calicatus* (7.4%), *Cor. coronatus* (5.2%) and *Cor. labratus* (0.7%). However, only six nematode species were observed in faecal DNA samples where resistance and/or suspected IVM resistance was detected at 2 weeks post-treatment. The two most abundant nematode species in IVM post-treatment samples were *Cylc. nassatus* (33.3%) and *Cyls. longibursatus* (22.8%), although species relative abundance varied among different samples (Fig. 2; Supplementary Table S5).

Eight nematode species were detected in four pre-treatment DNA samples for ABM groups. Ten species were detected in samples collected at 2 weeks post-treatment (Fig. 2; Supplementary Table S5). Similarly, twelve nematode species were detected in pre-treatment DNA samples in MOX groups (*n* = 14) which were reduced to eight species at 2 weeks post-treatment, with *Cor. coronatus* being the most abundant species (47.4%). A species of large strongyles, *T. brevicauda,* was also detected at 2 weeks post-treatment (Fig. 2; Supplementary Table S5).

Although resistance was not detected against ABM + MOR in 13 FECRTs at 2 weeks post-treatment, two species, including *Cya. catinatum* and *Cylc. nassatus* were identified at 2 weeks post-treatment. Both species were among 10 strongylid species identified in pre-treatment samples, with relative abundances of 29.8% and 1.7% for *Cylc. nassatus* and *Cya. catinatum*, respectively.

### 3.6 Nematode composition following treatment with OFZ and OFZ + PYR

Six nematode species were detected in pre-treatment samples in OFZ groups (*n* = 6), with the three most abundant species being *Cylc. nassatus* (62.3%), *Cyls. goldi* (14.8%) and *Cyls. longibursatus* (9.69%). In DNA samples collected 2 weeks post-treatment, eight species were detected (Fig. 2; Supplementary Table S6). Similarly, in 18 pre-treatment DNA samples for OFZ + PYR groups, 21 nematode species were identified, with the most abundant species being *Cylc. nassatus* (29.8%), *Cyls. longibursatus* (21.8%), *Cor. coronatus* (16.1%), *Cyls. minutus* (8.1%) and *Cylc. ashworthi* (8%). In addition, *T. nipponicus, T. serratus, T. brevicauda* and *Strongylus vulgaris* were detected in pre-treatment samples. While AR was detected in 15 out of 18 FECRTs, nine strongylid nematodes were detected in samples collected 2 weeks post-treatment. Surprisingly, in one test, *T. brevicauda* was also detected in 2 weeks post-treatment (Fig. 2; Supplementary Table S6).

### 3.7 Nematode composition at 4 and 5 weeks post-treatment

**Fig. 3:**
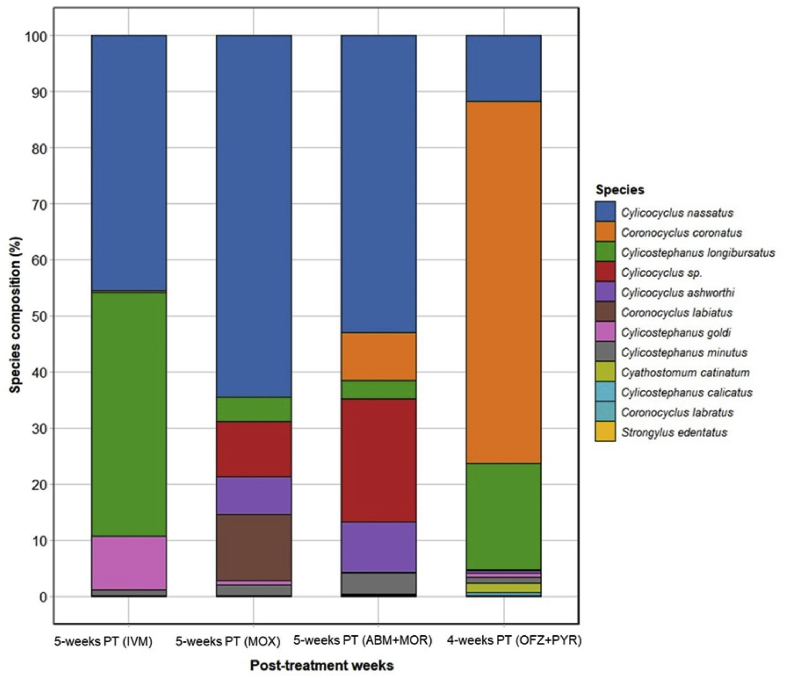
Relative abundance of strongylid nematodes from pooled faecal samples collected at 4- and 5 weeks post-treatment with various anthelmintics. Each taxon is defined by one colour. In each bar, the mean nemabiome of each taxon is based on the total number of variants of one species divided by the total number of counts of all species. Abbreviations: IVM, ivermectin; ABM, abamectin; MOX, moxidectin; OFZ, oxfendazole; FBZ, fenbendazole; PYR, pyrantel embonate; MOR, morantel tartrate.

Six nematode species were detected 5 weeks following treatment with IVM. Similarly, following treatments with MOX and ABM + MOR, eight and 10 nematode species were detected 5 weeks post-treatment, respectively (Fig. 3; Supplementary Table S5). *Cylc. nassatus* was the most abundant species detected at 5 weeks following treatments with MLs. Strongylid species composition following IVM and MOX treatment was similar except for three additional species *Cor. labratus, Cylc. ashworthi* and *Cylicocyclus* sp. which were detected in the MOX group whereas, *Strongylus edentatus* was detected at 5 weeks following IVM treatment. The nematode species composition following ABM and MOR treatment was similar to those detected for MOX groups at 5 weeks post-treatment, with the addition of *Cyls. calicatus* and *Cya. catinatum*. Eleven strongylid nematode species were detected at 4 weeks following treatment with OFZ + PYR where the species composition was similar to those of combinations of MLs at 5 weeks (Fig. 3; Supplementary Table S6). However, the OFZ + PYR group had the highest proportions of *Cor. coronatus* (64.6%) at 4 weeks post-treatment compared to *Cylc. nassatus* in the ML groups.

## 4 Discussion

To the best of our knowledge, this is the first comprehensive study to assess AR in ascarid and strongylid nematodes against commonly used anthelmintic in Australian Thoroughbred horses, where 86 FECRTs were performed on 22 farms across five states. This is also the first study globally where large-scale conventional and advanced molecular methods have been used to declare AR and determine strongylid species composition pre- and post-treatment, respectively. Resistance was observed in ascarid and strongylid nematodes to all available ML single active anthelmintics, including IVM, ABM and MOX, whereas resistance to OFZ and its combination with PYR was detected only in strongylids. We also calculated ERPs for anthelmintics where resistance was not detected at 2 weeks post-treatment. The shortened strongylid ERPs of 4–6 weeks were observed following treatment with all the drugs tested. The major species identified using PCR-NGS were from genera *Cylicostephanus*, *Cylicocyclus* and *Coronocyclus* genera. However, in some instances, a few additional species were detected at 2 weeks post-treatment. The most striking finding was the presence of *T. brevicauda* before and after treatment with OFZ + PYR. This is the first report suggesting the reduced efficacy of any product against large strongyles.

Despite this study's novelty and extensive scope, results should be carefully interpreted due to the following limitations. In a few FECRTs, the sample size was small (*n* = 5) and the FEC threshold for including animals in the test was low (≥30 EPG). However, this lower threshold of FECs was limited to a very small number of tests (*n* = 3/74). To reduce the number of DNA samples for testing using the PCR-directed NGS, we pooled the faecal samples after determining individual pre-treatment and post-treatment FECs based on horse age and anthelmintic treatment group at each farm per FECRT. Due to the pooling of samples and variations of EPG in horses, this method does not represent the nemabiome of individual horses within a treatment group as more than 15–20 strongylid species parasitise the horse intestine simultaneously, and each of these strongylid species produces an unequal number of eggs leading to uneven distribution of those in faeces. The small amount of each sample used in the pool might have allowed us to examine less than 1% of strongylid eggs found in horse faeces which may also have led to the underrepresentation of rare species. Although eggs were concentrated from the samples pool before collection for DNA extraction, the final volume (∼1 mL) stored for DNA extraction may not reflect the variation in EPGs and/or species composition. In assigning unique sequences of nematodes determined herein to particular genera and species, we used a pre-existing nemabiome library based on the ITS-2 sequences from previous nemabiome projects (https://www.nemabiome.ca) which could have led to the failure of a small proportion of identifications to species level due to genetic variation.

Resistance to multiple ML drugs, including IVM, ABM and MOX, was observed in cyathostomins in News South Wales and Victoria where most of the Thoroughbred horses are located in Australia. Multiple reports of cyathostomin resistance to MLs have been published in recent years globally (Nielsen, 2022), including Australia (Abbas et al., 2021), Brazil (Flores et al., 2020; Lignon et al., 2021; Martins et al., 2021), the UK (Bull et al., 2023) and the USA (Nielsen et al., 2020, 2022a). The growing evidence of AR to all available single active ML drugs is concerning as the indiscriminate use of MLs continues on Australian horse properties and elsewhere (Nielsen et al., 2018; Abbas et al., 2023a). For example, recent worm control practice surveys for Australian horse owners/farm managers and equine veterinarians revealed that MLs were the first choice of anthelmintics for preventative deworming of all age groups of horses (Wilkes et al., 2020; Abbas et al., 2023a). Moreover, the majority of breeders (∼70%) rely on regular interval-based deworming (Abbas et al., unpublished) with limited or no use of non-chemical-based control measures. The use of such a deworming strategy has also been reported previously at a majority of the Standardbred and Thoroughbred farms in Australia (Beasley et al., 2020; Wilkes et al., 2020), which is considered a primary contributing factor to the development of AR (Matthews, 2008; Sallé et al., 2017). Additionally, a low percentage (<30%) of farms were monitoring the efficacy of the dewormers used, thereby contributing to the risk of spreading AR among parasite and horse populations (Beasley et al., 2020; Abbas et al., unpublished). Another plausible cause for the development and spread of AR in the nematodes of Australian Thoroughbred horses on breeding properties is the frequent movement of horses across the country. A recent study by Nielsen et al. (2020) reported the first case of importation of ML-resistant cyathostomins from Ireland to the USA. Subsequently, one year later, resistance to the same anthelmintics was observed in horses born and bred in the USA (Nielsen et al., 2022a). Therefore, it is expected that resistant parasites can spread between different horse farms due to the movement of horses.

We did not observe resistance to the two combinations of the MLs with THPs (IVM + MOR, and IVM + PYR) which could be due to the additive effect of THPs. Previously, Scare et al. (2020) reported 100% efficacy of a combination of anthelmintics (MOX and oxibendazole) against resistant cyathostomin populations in the USA. However, the study population was never exposed to MOX, which presumably has preserved its efficacy against cyathostomins. A modelling study that simulated the development of AR showed that the use of combination products delayed its onset compared to using a single active when appropriate treatment strategies were applied (Scare et al., 2020). Currently, no anthelmintic containing a single active from the THP class is available for use in Australian horses which significantly decreases the use of these compounds and may prevent or delay resistance to this compound when used in combinations.

In this study, the resistance observed in cyathostomins to individual BZs (i.e., OFZ and FBZ) in eight FECRTs concurs with the findings of previous studies reporting widespread and well-established resistance to BZs (Rolfe and Dawson, 1994; Kuzmina and Kharchenko, 2008; Peregrine et al., 2014; Nielsen, 2022). Similarly, cyathostomin resistance to OFZ + PYR was found in 15 (out of 18) FECRTs at 2 weeks post-treatment and this reduction in FECs of animals treated with OFZ + PYR could be due to the effectiveness of PYR as anthelmintic products containing PYR alone are not available in Australia. However, resistance to a single active anthelmintics, BZs and THPs, have been consistently reported in cyathostomins worldwide (Traversa et al., 2012; Canever et al., 2013; Lester et al., 2013; Sallé et al., 2017; Bellaw et al., 2018; Morris et al., 2019; Butler et al., 2021; Zanet et al., 2021). In early 2000, a study reported treatment failure of MOR against strongyles in Australia on two properties (Pook et al., 2002). The status of AR to THPs is uncertain in Australia as we could not test the efficacy of PYR individually due to its unavailability. Based on the two recent worm control practice surveys for Australian horse owners/farm managers and equine veterinarians, OFZ + PYR was the second choice as a rotational dewormer for all age groups of horses (Abbas et al., 2023a; Abbas et al., unpublished). Widespread use of this combination could have selected strongylid nematodes for AR against these active ingredients. This supports the notion that combining two actives with reduced efficacies increases selection pressure for multi-drug resistance and may not be a sustainable practice for parasite control in horses. Scare et al. (2018) reported enhanced efficacy of a combination (oxibendazole + PYR) against cyathostomins while the individual anthelmintic efficacies were below effective limits, but the efficacy was not sustained in successive tests, suggesting that combination therapy against a double-resistant cyathostomin population would be unsustainable. In this study, we also found that OFZ + PYR was effective (i.e., %FECR ≥95%) on three farms of varying size (Farm 3: 27 horses, Farm 8: ∼100 horses and Farm 12: >500 horses), possibly because these drug classes had not been used in the last 2–3 years on these farms and parasite populations may not have been exposed to them and/or selected for resistance. However, the limited history of anthelmintic use available from the farm does not rule out the possibility of parasite prior exposure to these anthelmintics.

We found widespread resistance in *Parascaris* spp. to all available MLs, including ABM, IVM, and MOX in Australian horses. ML resistance in *Parascaris* spp. is well-established worldwide (Schougaard and Nielsen, 2007; von Samson-Himmelstjerna, 2012; Morris et al., 2019). Previously, resistance to single and/or multiple drugs, including ABM, FBZ, IVM and PYR, has been reported in Australia (Armstrong et al., 2014; Wilkes et al., 2017). However, in the current study, ABM + MOR and OFZ + PYR were still effective against *Parascaris* spp. These findings are consistent with a previous Australian study that reported resistance to anthelmintics containing a single active but ABM + MOR was 100% effective (Wilkes et al., 2017). Due to increased reports of *Parascaris* spp. resistance to single active ML compounds, the use of combination products containing more than one actives was suggested as a better strategy against this parasite (Geary et al., 2012; Kaplan et al., 2014). Such anthelmintic products might help diminish the rate of resistance development in *Parascaris* spp. due to the additive effect of anthelmintics (Kaplan et al., 2014).

Wherever the %FECR was below the effective limits at 2 weeks post-treatment, ERPs of 4–6 weeks were observed following treatment with MLs used as either a single active or combined with THPs. Similarly, reduced ERPs of 3, 4 and 6 weeks were observed following treatment with OFZ + PYR in three tests where AR was not detected at 2 weeks post-treatment. Other studies have reported shortened cyathostomin ERPs between 4 and 6 weeks following IVM (von Samson-Himmelstjerna et al., 2007; Geurden et al., 2014; Nielsen et al., 2022b) and MOX treatments (Rossano et al., 2010; Relf et al., 2014; van Doorn et al., 2014; Abbas et al., 2021). It has been proposed that shortened ERPs could be due to the selection pressure on larval stages of cyathostomins i.e., luminal L4s (LL4s) that survive the anthelmintic treatments, complete the last moult and start laying eggs and/or reduced efficacy of MLs against encysted stages of cyathostomins (Lyons et al., 2007; Lyons, 2009; Lyons and Tolliver, 2013). However, a shortened ERP for MOX (42 days) in a group of horses that were subsequently treated with PYR at 84 days post-treatment and determined to have an ERP within the expected range for PYR was reported (Kooyman et al., 2016a). The authors proposed that if the shortening of ERPs for MOX were attributable to the accelerated maturation of luminal stages, it would be observed independently of the anthelmintic used; hence, decreased MOX susceptibility was most likely the cause of a shortened ERP (Kooyman et al., 2016a). However, Nielsen et al. (2022b) suggested that LL4s may not be the only reason linked to the shortening of ERPs, as the low numbers of LL4s found two weeks post-treatment did not correspond with the thousands of adult stages recovered in IVM and MOX treated groups at the time of ERP, while the larvicidal efficacy of MOX was still within historical ranges. While it remains unknown whether the LL4s survived or appeared after treatment and contributed to reduced ERP, there are likely other reasons for an increased number of adult worms in the 5th week compared to the 2nd week post-treatment that was reported by Nielsen et al. (2022b). Another possible reason could be the appearance of certain species following treatment. For example, the appearance of *Cylicocyclus* sp., *Cya. catinatum* and *Cyls. longibursatus* (van Doorn et al., 2014; Kooyman et al., 2016a; Bellaw et al., 2018) following the shortening of the ERPs might be due to the selection of cyathostomin species with a shorter life cycle (Sangster, 1999; Geurden et al., 2014). *Cyls. longibursatus* has a prepatent period of 57 days and can reappear more quickly following deworming than other species with longer prepatent periods (Lyons et al., 2011). Previously, Kooyman et al. (2016a) found different species compositions following treatments with MOX and PYR and suggested that species with shorter prepatent periods develop more quickly from the mucosal stage to adult worms, leading to different species compositions. However, the prepatent periods of cyathostomins are known to change based on the age of horses and previous studies have made such estimations only in foals (Lyons et al., 2011). Further research is needed to understand the prepatent periods of various cyathostomins in young and adult horses.

We also found varied composition and/or relative abundance of nematode species at two weeks following treatment with different ML compounds. The predominant species identified at 2 weeks following IVM treatment were *Cylc. nassatus* (33.3%) and *Cyls. longibursatus* (22.8%) while those after treatment with MOX were *Cor. coronatus* (47.4%) and *Cyls. calicatus* (13.3%). Such patterns of species reappearance following treatment with MLs have been observed in a recent study from the USA where *Cya. catinatum* and *Cylc. nassatus* were the main species at 2 weeks following MOX treatment while *Cylc*. *insigne, Cylc. radiatus*, *Cya. catinatum, Cylc. elongatus* and *Cylc. nassatus* were species detected at two weeks following IVM treatment (Nielsen et al., 2022b). Additionally, these authors suggested differential efficacy between MOX and IVM for selected species as the former has larvicidal activity and may have reduced the efficacy against immature or developing larvae. Moreover, certain species may exhibit a greater propensity for adapting to specific climatic conditions which vary between the Northern and Southern hemispheres. Previously, it has been reported that the development and survival of strongyle larvae differ under cool-temperate (Northern hemisphere), warm-temperate (Southern hemisphere), and subtropical/tropical climates (Mfitilodze and Hutchinson, 1988; Hutchinson et al., 1989; Nielsen et al., 2007). For instance, *Cya. catinatum* is a species of high abundance and is among the three most abundant nematodes in most of the studies reported previously (Bellaw and Nielsen, 2020; Nielsen et al., 2022b; Sargison et al., 2022), but we found a very low prevalence of this nematode in the current study and previous studies (Abbas et al., 2023b). We also detected six additional strongylid species which were not detected pre-treatment (*Cyls. minutus*, *Cya. catinatum*, *Cyls. bidentatus, Cor. labiatus, Petrovinema poculatum* and *T. brevicauda*) but appeared two weeks following treatments with ABM and MOX. Most of the species that emerged after 2–5 weeks of treatment, which were absent in the pre-treatment samples, predominantly consisted of rare species previously identified in equines such as *Cyls. bidentatus, T. brevicauda* and *P.* *poculatum*. It is almost impossible to contaminate faecal samples with eggs/larvae of such extremely rare species from the ground (the proportion of these species in the strongylid community is less than 1%; thus, the number of their larvae in the environment is negligible). Additionally, the dilution effect resulting from the pooling samples for DNA extraction may have further reduced their proportions in the pooled sample, leading to decreased DNA yield. However, the higher sensitivity of the technique (i.e., NGS) likely enabled the detection of these species. In addition, the tested drugs were likely effective against the adult parasite populations, but their efficacy was reduced against the luminal L4s. The test used for nematode identification was based on the DNA extracted from parasite eggs, so it is possible that species from surviving LL4s with quicker development of this part of the life cycle were selected and contributed to egg laying at 2 weeks post-treatment and afterwards.

Although FECR was >95% at 2 weeks following treatment with ABM + MOR, *Cya. catinatum* and *Cylc. nassatus* were identified either due to the survival of a few adult worms (Nielsen et al., 2022b) or that of LL4s that started egg-laying after 2 weeks post-treatment. Similarly, where resistance to OFZ or its combination with PYR (OFZ+PYR) was observed, a subset of species present pre-treatment appeared again at 2 weeks post-treatment. However, two additional species (*Cyls. minutus* and *Cya. catinatum*) were also detected at 2 weeks post-OFZ-treatment. These results indicate the survival of adult worms following treatment with OFZ + PYR which continue to shed eggs at 2 weeks post-treatment.

Similar strongylid species compositions were detected following IVM and MOX at 5 weeks post-treatment. However, *Cor. labratus, Cylc. ashworthi* and *Cylicocyclus* sp. were also detected in the MOX group. The appearance of *S*. *edentatus* in the IVM-treated group at 5 weeks post-treatment was remarkable. We found treatment failure against another species of large strongyle (i.e., *T. brevicauda*) at 2 weeks post-treatment and the appearance of *S. edentatus* at 5 weeks highlights the concern that current treatment protocols used to control strongylid nematodes can select species of pathogenic large strongyles for AR in the future. Similarly, two additional species (*Cyls. calicatus* and *Cya. catinatum*) were observed at 5 weeks post-treatment with ABM + MOR. Regarding the combination of BZs and THPs, *Cor. coronatus* had the highest proportions at 5 weeks post-treatment with OFZ and PYR compared to *Cylc. nassatus* in the case of MLs and combinations of ML and THPs. A number of reasons have been suggested for the shortening of ERPs of cyathostomins (Macdonald et al., 2023; Nielsen et al., 2023). For example, it is important to note that prepatent periods for cyathostomins are not well-defined. Anthelmintic treatments would likely have selected the cyathostomin species with shorter prepatent periods to resume development following deworming, leading to the reduced ERPs for anthelmintics used. Previously, the prepatent period for *Cor. coronatus* was estimated to be 70 days in foals (Lyons et al., 2011), but it is unclear if this is similar in adult horses due to potential acquired immunity against GINs. It is possible that frequent use of OFZ + PYR might have selected for resistance in *Cor. coronatus* as it has been found in higher proportions in Australia (Abbas et al., 2023b) compared to the Northern hemisphere where this nematode species is of medium abundance (Bellaw and Nielsen, 2020). However, more evidence is required to definitively determine this. Additional studies are required to investigate possible associations between reduced ERPs and the resumption of encysted larval development, cyathostomin species composition and their prepatent periods in different age groups of horses.

In this study, horses were maintained on pastures. Most of the participating Thoroughbred farms were using frequent, prophylactic interval-based deworming which could have contributed to the variation in the composition of strongylid nematodes detected. Previously, differences in nematode species composition were observed on various farms based on differences in anthelmintic treatment strategies (Kuzmina and Kharchenko, 2008). Furthermore, the frequent use of anthelmintics was associated with a higher abundance of common species (*n* = 10–12) while rare species were more prevalent in horses that received no or fewer anthelmintic treatments (Kuzmina et al., 2016). It is also important to note that the variation in reported strongylid nematodes could also be due to the variation in diagnostic methods. Most studies have used coproculture, necropsy or morphological identification of naturally expelled parasites following anthelmintic treatments. However, some studies have used molecular tools such as reverse line blot assays which require species-specific markers that may not be available for all the nematode species that infect horses (Kooyman et al., 2016b).

Recently, Poissant et al. (2021) demonstrated that ITS-2 DNA metabarcoding led to a better understanding of the nematode diversity than traditional coprological methods (Poissant et al., 2021). While the identification of adult worms at necropsy remains the gold standard test in horses or other livestock species, a recent study demonstrated a concordance between the results of DNA metabarcoding and morphological identification of nematodes in horses (Nielsen et al., 2022b). DNA metabarcoding represents a non-invasive approach that facilitates the identification of prevalent strongylid species exhibiting AR or shortened ERPs. Therefore, incorporating DNA metabarcoding into future studies on FECRTs or ERPs would provide valuable insights into underlying reasons for AR development and/or shortening of ERPs. Furthermore, this method could contribute to a better understanding of the association between various factors, such as climate and farming practices. and the development of AR and shortening of ERPs in specific strongylid species for various anthelmintics used in horses. This would lead to developing comprehensive information on any species that developed resistance to a particular anthelmintic(s). Comparisons of such details are more convenient to draw a meaningful conclusion if the FECRT methods follow a uniform design. Previously, equine-specific criteria for FECRTs were not well-established. However, the new WAAVP guidelines provide comprehensive details about all aspects of efficacy study designs, from sampling, statistical powers to interpretations (Kaplan et al., 2023). In the current study, we could not implement the latest WAAVP guidelines (Kaplan et al., 2023) in the study design as the study commenced in 2020. Nevertheless, we conducted a statistical power analysis for the FECRT using generated data and applied the new (research) thresholds to assess drug efficacy. Notably, there was a broad consensus between the two methods, with resistance calculated using the old approach being confirmed by the new thresholds. Furthermore, the application of the new thresholds revealed the identification of resistance in six additional tests for strongyles and three more tests for *Parascaris* spp. In the case of strongylids, this occurred when suspected resistance (*n* = 3) was already found using the old guidelines, while new resistance profiles emerged from susceptibility for *Parascaris* spp. These findings were expected, considering the more rigorous criteria of the new guidelines based on expected and lower efficacy thresholds for each drug class. However, it is important to interpret the results obtained using the new efficacy thresholds cautiously, as the study may not always fully comply with the new guidelines in terms of study design. For instance, there may be a smaller number of horses included and/or less egg counted in the tests, which could have influenced the outcomes.

## 5 Conclusion

This study provides information about the status of AR in cyathostomins and *Parascaris* spp. against commonly used anthelmintics in Australian Thoroughbred horses, with the first report of the apparent failure of OFZ + PYR treatment against *T. brevicauda*, a species of large strongyle. Cyathostomins were found resistant to multiple anthelmintics, including single actives such as ABM, IVM, MOX and OFZ, and combination products (OFZ + PYR). Similarly, resistance to IVM, ABM and MOX was detected against *Parascaris* spp., and when data were analysed using new research thresholds (Kaplan et al., 2023), resistance was also observed to OFZ and its combination with PYR. In general, there was good agreement between the two methods, with resistance calculated using the old criteria being confirmed by the new thresholds. Furthermore, the application of the new thresholds revealed the identification of resistance in six additional tests for strongyles and three more tests for *Parascaris* spp. The ERPs of cyathostomins following various anthelmintics were reduced to four and/or six weeks. In most of the FECRTs where resistance to a particular compound was observed, the nematode species composition showed homogeneity in pre- and post-treatment samples. The major cyathostomin species identified at 2 weeks post-treatment were from two genera, *Cylicocyclus* and *Cylicostephanus*. The major cyathostomins appearing at five weeks following treatment with MLs were *Cylc. nassatus, Cyls. longibursatus* and *Cylc. ashworthi*. In the case of OFZ + PYR, two additional species (*Cor. coronatus* and *Cya. catinatum*) were found. These findings provide information about the current status of AR and its patterns in strongylid nematodes and *Parascaris* spp. on Australian Thoroughbred farms. This study also highlights the need to change the current deworming practices used on Australian Thoroughbred farms and regularly monitor the efficacy of available anthelmintics to manage AR.

## Funding

This work was supported by AgriFutures Australia, Thoroughbred Breeders Australia and 10.13039/100001003Boehringer Ingelheim, Australia. The funding partners did not have any role in the design or content of this study. The PhD student, Ghazanfar Abbas, is a grateful recipient of the Australian Government Research Training Scholarship through the University of Melbourne.

## Declaration of competing interest

The authors of this manuscript are members of the Australian Equine Parasitology Advisory Panel (AEPAP), including Abdul Jabbar, Ghazanfar Abbas, Jenni Bauquier, Charles El-Hage, Abdul Ghafar and Ian Beveridge (The University of Melbourne), Anne Beasley (University of Queensland), Kristopher Hughes (Charles Sturt University), Caroline Jacobson and Emma McConnell (Murdoch University), Edwina Wilkes (Racing Victoria), Peter Carrigan and Lucy Cudmore (Scone Equine Hospital) and John Hurley (Swettenham Stud). 10.13039/100001003Boehringer Ingelheim supported the panel.

The authors declare that they have no known competing financial interests or personal relationships that could have appeared to influence the work reported in this paper.
