# Supplementary material for: A national survey of anthelmintic resistance in ascarid and strongylid nematodes in Australian Thoroughbred horses
Source: Int J Parasitol Drugs Drug Resist. 2023 Nov 29;24:100517. doi: 10.1016/j.ijpddr.2023.11.006 (PMC10757041; doi:10.1016/j.ijpddr.2023.11.006)
Supplement: Multimedia component 1 [file mmc1.zip › Revised Ms. IJPDDR-D-23-00067_R2_Supplementary Tables.DOCX]

Supplementary Table 1. Efficacy of treatments with macrocyclic lactones against strongylid nematodes in Thoroughbred horses across Australia.

| Drugs used and farm codes | State | Age group | No. of animals included in test | Total eggs counted (Pre-T) | Mean faecal egg counts (eggs per gram) | | % FECR (95% credible intervals) | Test outcome | 90% credible intervals * | | Efficacy classification | Required sample size ** |
| --- | --- | --- | --- | --- | --- | --- | --- | --- | --- | --- | --- | --- |
|  |  |  |  |  | Pre-treatment | 2 weeks post-treatment |  |  | Lower | Upper |  |  |
| **Ivermectin** | | | | | | | | | | | |  |
| 1 | NSW | W | 10 | 1169 | 1717 | 303 | 82 (80-85) | **Resistant** | 73 | 90.2 | Resistant | 5 |
| 3 | NSW | Y | 6 | 496 | 1232 | 2 | 100 (99-100) | Susceptible | 97.9 | 99.7 | Low resistant | 5 |
| 6 | QLD | Y | 7 | 362 | 802 | 15 | 98 (97-99) | Susceptible | 96.2 | 99.7 | Low resistant | 6 |
| 8 | WA | Y | 7 | 396 | 877 | 10 | 99 (98-100) | Susceptible | 97.5 | 99.8 | Low resistant | 6 |
| 9 | VIC | M | 9 | 471 | 818 | 1 | 100 (99-100) | Susceptible | Uncalculable | | Susceptible | 6 |
| 10 | VIC | M | 13 | 844 | 985 | 4 | 100 (99-100) | Susceptible | 98.9 | 100 | Susceptible | 5 |
| 11 | VIC | Y | 10 | 1028 | 1515 | 86 | 94 (93-96) | **Suspected resistance** | 82.5 | 99.8 | Resistant | 5 |
| 13 | NSW | W | 7 | 817 | 1703 | 6 | 100 (99-100) | Susceptible | 99.3 | 100 | Susceptible | 5 |
| 13 | NSW | Y | 7 | 456 | 989 | 71 | 93 (90-95) | **Resistant** | 76.8 | 99.8 | Resistant | 5 |
| 14 | NSW | Y | 15 | 1563 | 1535 | 23 | 99 (98-99) | Susceptible | 95.7 | 99.9 | Inconclusive | 5 |
| 15 | NSW | Y | 12 | 959 | 1204 | 5 | 100 (99-100) | Susceptible | 99 | 100 | Susceptible | 5 |
| 16 | QLD | Y | 9 | 675 | 1133 | 1 | 100 (100-100) | Susceptible | Uncalculable | | Susceptible | 6 |
| 17 | VIC | W | 13 | 157 | 193 | 1 | 100 (98-100) | Susceptible | Uncalculable | | Susceptible | 12 |
| 18 | VIC | Y | 12 | 119 | 155 | 1 | 100 (98-100) | Susceptible | Uncalculable | | Susceptible | 14 |
| 18 | VIC | W | 15 | 1290 | 1305 | 1 | 100 (99-100) | Susceptible | 99 | 99.8 | Low resistant | 5 |
| **Abamectin** | | | | | | | | | | | |  |
| 12 ^a^ | VIC | W | 5 | 258 | 810 | 220 | 73 (65-79) | **Resistant** | 39.6 | 94.3 | Resistant | 6 |
| 21 | NSW | Y | 12 | 704 | 887 | 69 | 92 (90-94) | **Suspected resistance** | 87.4 | 96.2 | Resistant | 6 |
| 21 | NSW | M | 9 | 295 | 526 | 42 | 92 (88-95) | **Resistant** | 81.6 | 98.6 | Resistant | 7 |
| 22 | SA | Y | 6 | 572 | 1407 | 63 | 96 (94-97) | Susceptible | 90.3 | 99 | Resistant | 5 |
| **Moxidectin** | | | | | | | | | | | |  |
| 4 | NSW | Y | 10 | 1032 | 1507 | 109 | 93(91-94) | **Suspected resistance** | 79.3 | 99.5 | Resistant | 5 |
| 5 | NSW | W | 13 | 675 | 802 | 69 | 91 (89-94) | **Resistant** | 77.2 | 99.2 | Resistant | 6 |
| 6 | QLD | Y | 7 | 821 | 1721 | 8 | 100 (99-100) | Susceptible | 98.8 | 100 | Susceptible | 5 |
| 8 | WA | Y | 7 | 851 | 1768 | 8 | 100 (99-100) | Susceptible | 99.2 | 99.9 | Susceptible | 5 |
| 12 ^a^ | VIC | W | 5 | 182 | 511 | 3 | 100 (98-100) | Susceptible | Uncalculable | | Susceptible | 7 |
| 12 ^a^ | VIC | Y | 5 | 160 | 579 | 2 | 100 (99-100) | Susceptible | Uncalculable | | Susceptible | 7 |
| 13 | NSW | Y | 7 | 331 | 758 | 11 | 99 (97-100) | Susceptible | 96.7 | 99.9 | Low resistant | 6 |
| 14 | NSW | Y | 15 | 1719 | 969 | 4 | 100 (99-100) | Susceptible | 99.3 | 99.9 | Susceptible | 5 |
| 15 | NSW | Y | 15 | 1104 | 1114 | 2 | 100 (100-100) | Susceptible | 99.7 | 100 | Susceptible | 5 |
| 18 | VIC | Y | 14 | 586 | 643 | 1 | 100 (100-100) | Susceptible | Uncalculable | | Susceptible | 7 |
| 18 | VIC | W | 15 | 985 | 1004 | 4 | 100 (100-100) | Susceptible | 99.4 | 99.9 | Susceptible | 5 |
| 19 | NSW | Y | 16 | 949 | 1065 | 5 | 100 (100-100) | Susceptible | 99.2 | 100 | Susceptible | 5 |
| 20 ^a^ | VIC | W | 5 | 347 | 1053 | 120 | 89 (85-92) | **Resistant** | 83% | 93.5 | Resistant | 5 |
| 20 ^a^ | VIC | Y | 7 | 714 | 1477 | 143 | 90 (88-93) | **Resistant** | 77.4 | 98.2 | Resistant | 5 |

NSW, New South Wales; QLD, Queensland; SA, South Australia; VIC, Victoria; WA, Western Australia; %FECR, percent faecal egg count reduction; Y, yearlings; W, weanlings; M, mares; the outcome of FECRT was declared as susceptible, resistant, or suspected resistance based on Coles et al. (1992).

^a^ Samples included from the previous study (Abbas et al., 2021).

* Based on new WAAVP guidelines (Kaplan et al., 2023: expected efficacy of 99.9% and a lower efficacy threshold of 96%), calculated using open source bayescount package, version 1.1.0 via <https://www.fecrt.com>. In tests where FECs were all zero at post treatment, the 90% CI were ‘Uncalculable’ and were categorised as susceptible.

** Required sample size for a faecal egg count reduction test as per new WAAVP guidelines (Kaplan et al., 2023), calculated using open source bayescount package, version 1.1.0 via <https://www.fecrt.com>.

Supplementary Table 2. Efficacy of treatments with combinations of macrocyclic lactones and tetrahydropyridines against strongylid nematodes in Thoroughbred horses across Australia.

| Drugs used and farm codes | State | Age group | No. of animals included in test | Total eggs counted (Pre-T) | Mean faecal egg counts (eggs per gram) | | % FECR (95% credible intervals) | Test outcome | 90% credible intervals * | | Efficacy classification | Required sample size ** |
| --- | --- | --- | --- | --- | --- | --- | --- | --- | --- | --- | --- | --- |
|  |  |  |  |  | Pre-treatment | 2 weeks post-treatment |  |  | Lower | Upper |  |  |
| **Abamectin and morantel tartrate ^¥^** | | | | | | | | | | | |  |
| 1 | NSW | Y | 12 | 946 | 1189 | 2.1 | 100 (99-100) | Susceptible | 99.7 | 100 | Susceptible | 5 |
| 2 | NSW | Y | 21 | 3032 | 2114 | 1 | 100 (100-100) | Susceptible | Uncalculable | | Susceptible | 5 |
| 4 | NSW | Y | 10 | 1095 | 1616 | 32 | 98 (97-99) | Susceptible | 93.9 | 99.9 | Inconclusive | 5 |
| 5 | NSW | W | 14 | 736 | 827 | 1 | 100 (100-100) | Susceptible | Uncalculable | | Susceptible | 6 |
| 6 | QLD | W | 6 | 133 | 373 | 13 | 97 (93-99) | Susceptible | 93.2 | 99.4 | Resistant | 9 |
| 12 ^a^ | VIC | W | 5 | 54 | 201 | 3 | 100 (94-100) | Susceptible | Uncalculable | | Susceptible | 12 |
| 12 ^a^ | VIC | W | 5 | 187 | 616 | 2 | 100 (98-100) | Susceptible | Uncalculable | | Susceptible | 7 |
| 13 | NSW | W | 8 | 1104 | 1996 | 1 | 100 (100-100) | Susceptible | Uncalculable | | Susceptible | 5 |
| 13 | NSW | Y | 5 | 401 | 1187 | 2 | 100 (99-100) | Susceptible | Uncalculable | | Susceptible | 5 |
| 15 | NSW | Y | 15 | 1479 | 1475 | 33 | 98 (97-99) | Susceptible | 93.1 | 99.9 | Inconclusive | 5 |
| 17 | VIC | W | 19 | 1108 | 886 | 1 | 100 (100-100) | Susceptible | 99.7 | 100 | Susceptible | 6 |
| 19 | NSW | Y | 15 | 1507 | 1498 | 1 | 100 (100-100) | Susceptible | Uncalculable | | Susceptible | 5 |
| 20 | VIC | F | 7 | 1227 | 2342 | 1 | 100 (100-100) | Susceptible | Uncalculable | | Susceptible | 5 |
| **Ivermectin and pyrantel embonate ^¥^** | | | | | | | | | | | |  |
| 4 | NSW | Y | 10 | 1094 | 1608 | 1 | 100 (100-100) | Susceptible | Uncalculable | | Susceptible | 5 |
| 13 | NSW | W | 8 | 648 | 1210 | 1 | 100 (100-100) | Susceptible | Uncalculable | | Susceptible | 5 |

NSW, New South Wales; QLD, Queensland; VIC, Victoria; %FECR, percent faecal egg count reduction; Y, yearlings; W, weanlings; F, foals; the outcome of FECRT was declared as susceptible, resistant, or suspected resistance based on Coles et al. (1992).

^a^ Samples included from the previous study (Abbas et al., 2021).

* Based on new WAAVP guidelines (Kaplan et al., 2023: expected efficacy of 99.9% and a lower efficacy threshold of 96%), calculated using open source bayescount package, version 1.1.0 via <https://www.fecrt.com>. In tests where FECs were all zero at post treatment, the 90% CI were ‘Uncalculable’ and were categorised as susceptible.

** Required sample size for a faecal egg count reduction test as per new WAAVP guidelines (Kaplan et al., 2023), calculated using open source bayescount package, version 1.1.0 via <https://www.fecrt.com>.

^¥^ For combination products, we used the higher efficacy thresholds of the individual drug used in the combination.

Supplementary Table 3. Efficacy of treatments with benzimidazole and their combinations with tetrahydropyridines against strongylid nematodes in Thoroughbred horses across Australia.

| Drugs used and farm codes | State | Age group | No. of animals included in each test | Total eggs counted (Pre-T) | Mean faecal egg counts (EPG) | | % FECR (95% credible intervals) | Test outcome | 90% credible intervals * | | Efficacy classification | Required sample size ** |
| --- | --- | --- | --- | --- | --- | --- | --- | --- | --- | --- | --- | --- |
|  |  |  |  |  | Pre-treatment | 2 weeks post-treatment |  |  | Lower | Upper |  |  |
| **Oxfendazole** | | | | | | | | | | | |  |
| 5 | NSW | W | 14 | 822 | 959 | 887 | 7 (0-14) | **Resistant** | -58.6 | 56.8 | Resistant | 11 |
| 6 | QLD | W | 5 | 143 | 461 | 394 | 14 (0-28) | **Resistant** | -34.3 | 50.7 | Resistant | 14 |
| 6 | QLD | Y | 6 | 678 | 1596 | 987 | 38 (31-46) | **Resistant** | -13.3 | 76.1 | Resistant | 10 |
| 12 | VIC | W | 5 | 20 | 69 | 33 | 56 (13-81) | **Resistant** | 9.2 | 86.2 | Resistant | No result |
| 12 ^a^ | VIC | W | 5 | 265 | 990 | 974 | 0 (0-5) | **Resistant** | -218 | 69.6 | Resistant | 11 |
| 13 | NSW | Y | 6 | 41 | 103 | 473 | 0 (0-4) | **Resistant** | -785 | -61.3 | Resistant | No result |
| 20 | VIC | W | 5 | 266 | 890 | 841 | 5 (0-15) | **Resistant** | -118 | 74.5 | Resistant | 11 |
| **Fenbendazole** | | | | | | | | | | | |  |
| 15 | NSW | Y | 12 | 1081 | 1340 | 717 | 47 (41-52) | **Resistant** | -24.4 | 89.8 | Resistant | 10 |
| **Oxfendazole and pyrantel embonate ^¥^** | | | | | | | | | | | |  |
| 1 | NSW | Y | 12 | 1744 | 2128 | 1229 | 42 (37-47) | **Resistant** | 18.2 | 62.7 | Resistant | 9 |
| 2 | NSW | W | 11 | 1513 | 1929 | 536 | 72 (69-75) | **Resistant** | 47.2 | 90.1 | Resistant | 9 |
| 3 | NSW | F | 7 | 183 | 427 | 2 | 100 (98-100) | Susceptible | Uncalculable | | Susceptible | 14 |
| 4 | NSW | Y | 8 | 1104 | 1869 | 503 | 73 (69-76) | **Resistant** | 46.6 | 91.5 | Resistant | 9 |
| 5 | NSW | W | 14 | 1663 | 1751 | 1,531 | 12 (5-18) | **Resistant** | -27.7 | 46.2 | Resistant | 10 |
| 6 | QLD | Y | 9 | 730 | 1206 | 984 | 19 (9-27) | **Resistant** | -54.7 | 70.8 | Resistant | 10 |
| 7 | WA | Y | 12 | 1201 | 1493 | 1,222 | 18 (11-25) | **Resistant** | -15.4 | 46.6 | Resistant | 10 |
| 8 | WA | Y | 6 | 114 | 322 | 16 | 95 (90-98) | Susceptible | 90.1 | 99.1 | Inconclusive | 16 |
| 9 | VIC | W | 12 | 1485 | 1818 | 592 | 68 (64-71) | **Resistant** | 49.1 | 82.4 | Resistant | 10 |
| 10 | VIC | Y | 18 | 1826 | 1505 | 304 | 80 (77-82) | **Resistant** | 67.6 | 89.5 | Resistant | 10 |
| 11 | VIC | Y | 11 | 929 | 1271 | 242 | 81 (78-84) | **Resistant** | 67.3 | 91.5 | Resistant | 10 |
| 12 ^a^ | VIC | W | 5 | 290 | 901 | 169 | 82 (76-86) | **Resistant** | 64.8 | 93.7 | Resistant | 11 |
| 12 | VIC | W | 5 | 147 | 491 | 26 | 95 (91-98) | Susceptible | 89.8 | 98.8 | Resistant | 13 |
| 13 | NSW | Y | 7 | 389 | 834 | 1,228 | 0 (0-3) | **Resistant** | -129.4 | 18.8 | Resistant | 11 |
| 13 | NSW | W | 8 | 176 | 330 | 709 | 0 (0-3) | **Resistant** | -269.6 | 2.8 | Resistant | 16 |
| 14 | NSW | W | 10 | 1511 | 2203 | 1,231 | 44 (39-49) | **Resistant** | 13 | 69.4 | Resistant | 9 |
| 14 | NSW | Y | 7 | 540 | 1171 | 582 | 50 (43-57) | **Resistant** | 15.6 | 77.2 | Resistant | 10 |
| 20 | VIC | W | 7 | 88 | 301 | 294 | 8 (0-7) | **Resistant** | -268.7 | 46.5 | Resistant | 16 |

NSW, New South Wales; QLD, Queensland; VIC, Victoria; WA, Western Australia; %FECR, percent faecal egg count reduction; Y, yearlings; W, weanlings; F, foals; the outcome of FECRT was declared as susceptible, resistant, or suspected resistance based on Coles et al. (1992).

^a^ Samples included from the previous study (Abbas et al., 2021).

* Based on new WAAVP guidelines (Kaplan et al., 2023: expected efficacy of 99% and a lower efficacy threshold of 95%), calculated using open source bayescount package, version 1.1.0 via <https://www.fecrt.com>. In tests where FECs were all zero at post treatment, the 90% CI were ‘Uncalculable’ and were categorised as susceptible.

^¥^ For combination products, we used the higher efficacy thresholds of the individual drug used in the combination.

** Required sample size for a faecal egg count reduction test as per new WAAVP guidelines (Kaplan et al., 2023), calculated using open source bayescount package, version 1.1.0 via <https://www.fecrt.com>.

Supplementary Table 4. Efficacy of single and combinations of anthelmintic drugs against ascarid nematodes in Thoroughbred horses across Australia.

| Drugs used and farm codes | State | Age group | No. of animals included in each test | Total eggs counted (Pre-T) | Mean faecal egg counts (EPG) | | % FECR (95% credible intervals) | Test outcome | 90% credible intervals * | | Efficacy classification | Required sample size ** |
| --- | --- | --- | --- | --- | --- | --- | --- | --- | --- | --- | --- | --- |
|  |  |  |  |  | Pre-treatment | 2 weeks post-treatment |  |  | Lower | Upper |  |  |
| **Ivermectin** | | | | | | | | | | | |  |
| 13 | NSW | W | 6 | 193 | 553 | 493 | 10 (0-23) | **Resistant** | -145.5 | 90.6 | Resistant | 5 |
| 17 | VIC | W | 13 | 503 | 663 | 25 | 96 (94-98) | Susceptible | 88.8 | 99.9 | Resistant | 5 |
| 18 | VIC | F | 8 | 881 | 1599 | 928 | 43 (36-48) | **Resistant** | -4.9 | 76.8 | Resistant | 5 |
| **Abamectin** | | | | | | | | | | | |  |
| 21 | NSW | Y | 6 | 99 | 521 | 511 | 0 (0-5) | **Resistant** | -615.7 | 94.3 | Resistant | 6 |
| **Moxidectin** | | | | | | | | | | | |  |
| 20 | VIC | F | 5 | 81 | 237 | 648 | 0 (0-0.4) | **Resistant** | -630.7 | 76 | Resistant | 9 |
| **Oxfendazole** | | | | | | | | | | | |  |
| 12 | VIC | F | 5 | 142 | 463 | 28 | 94 (89-97) | Susceptible | 82.8 | 99.7 | Resistant | 6 |
| **Fenbendazole** | | | | | | | | | | | |  |
| 18 | VIC | F | 6 | 439 | 1106 | 2 | 100 (99-100) | Susceptible | Uncalculable | | Susceptible | 5 |
| **Oxfendazole and pyrantel embonate** | | | | | | | | | | | |  |
| 3 | NSW | F | 7 | 947 | 1,825 | 21 | 99 (98-99) | Susceptible | 96.7 | 100 | Susceptible | 5 |
| 9 | VIC | W | 12 | 196 | 286 | 1 | 100 (98-100) | Susceptible | Uncalculable | | Susceptible | 8 |
| 10 | VIC | W | 9 | 331 | 597 | 14 | 98 (96-99) | Susceptible | 92.8 | 100 | Inconclusive | 5 |
| 12 | VIC | F | 5 | 302 | 939 | 39 | 96 (93-98) | Susceptible | 91.6 | 98.9 | Resistant | 5 |
| **Abamectin and morantel tartrate** | | | | | | | | |  |  |  |  |
| 12 | VIC | F | 5 | 461 | 1367 | 2 | 100 (99-100) | Susceptible | Uncalculable | | Susceptible | 5 |

NSW, New South Wales; VIC, Victoria; %FECR, percent faecal egg count reduction; Y, yearlings; W, weanlings; F, foals; the outcome of FECRT was declared as susceptible, resistant, or suspected resistance based on Coles et al. (1992).

* Based on new WAAVP guidelines (Kaplan et al., 2023: expected efficacy of 99.9% and a lower efficacy threshold of 95%), calculated using open source bayescount package, version 1.1.0 via <https://www.fecrt.com>. In tests where FECs were all zero at post treatment, the 90% CI were ‘Uncalculable’ and were categorised as susceptible.

** Required sample size for a faecal egg count reduction test as per new WAAVP guidelines (Kaplan et al., 2023), calculated using open source bayescount package, version 1.1.0 via <https://www.fecrt.com>.

Supplementary Table 5. Strongylid species composition (%) before and after treatment with macrocyclic lactones and their combinations in Thoroughbred horses across Australia.

| Anthelmintics used | Strongylid species | Counts (%) | | |
| --- | --- | --- | --- | --- |
|  |  | Pre-treatment | 2-week post-treatment | 5-week post-treatment |
| Ivermectin |  |  |  |  |
|  | *Cylicostephanus longibursatus* | 30.6 | 22.8 | 43.6 |
|  | *Cylicocyclus nassatus* | 26.6 | 33.3 | 45.6 |
|  | *Triodontophorus brevicauda* | 13.5 | 0 | 0 |
|  | *Cylicocyclus* sp. | 10.1 | 19.2 | 0 |
|  | *Cylicocyclus insigne* | 5.14 | 0 | 0 |
|  | *Cylicostephanus goldi* | 3.33 | 0 | 9.54 |
|  | *Cylicostephanus calicatus* | 2.34 | 5.8 | 0 |
|  | *Cylicostephanus minutus* | 2.25 | 0 | 1.1 |
|  | *Cyathostomum catinatum* | 1.92 | 0 | 0 |
|  | *Cylicocyclus ashworthi* | 1.64 | 17.1 | 0 |
|  | *Coronocyclus labiatus* | 1.55 | 0 | 0 |
|  | *Coronocyclus labratus* | 0.458 | 0 | 0 |
|  | *Strongylus edentatus* | 0.249 | 0 | 0.03 |
|  | *Strongylus vulgaris* | 0.191 | 0 | 0 |
|  | *Poteriostomum imparidentatum* | 0.123 | 0 | 0 |
|  | *Cylicostephanus* sp. | 0 | 1.9 | 0 |
|  | *Coronocyclus coronatus* | 0 | 0 | 0.2 |
| Abamectin |  |  |  |  |
|  | *Cylicocyclus nassatus* | 47.7 | 14.2 | NA |
|  | *Coronocyclus coronatus* | 37.6 | 17.5 | NA |
|  | *Cylicostephanus longibursatus* | 8.7 | 0 | NA |
|  | *Cylicostephanus goldi* | 2.1 | 0 | NA |
|  | *Cylicocyclus* sp. | 1.2 | 0 | NA |
|  | *Cylicostephanus calicatus* | 1.2 | 0.8 | NA |
|  | *Coronocyclus labratus* | 0.388 | 2.5 | NA |
|  | *Poteriostomum imparidentatum* | 0.272 | 1.2 | NA |
|  | *Cylicostephanus minutus* | 0 | 36.8 | NA |
|  | *Cyathostomum catinatum* | 0 | 15.4 | NA |
|  | *Cylicostephanus bidentatus* | 0 | 6.5 | NA |
|  | *Coronocyclus labiatus* | 0 | 3.1 | NA |
|  | *Petrovinema poculatum* | 0 | 0.1 | NA |
| Moxidectin |  |  |  |  |
|  | *Cylicocyclus nassatus* | 43.4 | 12.3 | 64.6 |
|  | *Cylicostephanus longibursatus* | 29 | 3.1 | 4.3 |
|  | *Coronocyclus coronatus* | 9.34 | 47.4 | 0 |
|  | *Cylicocyclus ashworthi* | 6.17 | 0 | 6.7 |
|  | *Cylicostephanus goldi* | 3.84 | 12.4 | 0.8 |
|  | *Cylicocyclus* sp. | 3.53 | 3.4 | 9.7 |
|  | *Cylicostephanus calicatus* | 1.63 | 13.3 | 0 |
|  | *Cyathostomum pateratum* | 1.02 | 0 | 0 |
|  | *Cylicostephanus minutus* | 0.957 | 4.2 | 1.9 |
|  | *Coronocyclus labratus* | 0.578 | 0 | 0.1 |
|  | *Poteriostomum imparidentatum* | 0.383 | 0 | 0 |
|  | *Cylicostephanus* sp. | 0.15 | 0 | 0 |
|  | *Triodontophorus brevicauda* | 0 | 3.34 | 0 |
|  | *Coronocyclus labiatus* | 0 | 0 | 11.2 |
| Abamectin + Morantel tartrate |  |  |  |  |
|  | *Cylicostephanus longibursatus* | 32.7 | 0 | 3.2 |
|  | *Cylicocyclus nassatus* | 29.8 | 26 | 53 |
|  | *Coronocyclus coronatus* | 13.8 | 0 | 8.6 |
|  | *Cylicostephanus goldi* | 9.39 | 0 | 0.1 |
|  | *Cylicostephanus minutus* | 3.46 | 0 | 8.9 |
|  | *Cylicostephanus calicatus* | 3.03 | 0 | 0.3 |
|  | *Cylicocyclus* sp. | 2.67 | 0 | 22.1 |
|  | *Cylicocyclus ashworthi* | 2.06 | 0 | 8.9 |
|  | *Cyathostomum catinatum* | 1.65 | 74 | 0.006 |
|  | *Coronocyclus labratus* | 1.36 | 0 | 0.02 |

Supplementary Table 6. Strongylid species composition (%) before and after treatment with benzimidazole and their combinations in Thoroughbred horses across Australia.

| Anthelmintics used | Strongylid species | Counts (%) | | |
| --- | --- | --- | --- | --- |
|  |  | Pre-treatment | 2-week post-treatment | 4-week post-treatment |
| Oxfendazole |  |  |  |  |
|  | *Cylicocyclus nassatus* | 62.3 | 50.5 | NA |
|  | *Cylicostephanus goldi* | 14.8 | 7.5 | NA |
|  | *Cylicostephanus longibursatus* | 9.69 | 17 | NA |
|  | *Cylicostephanus calicatus* | 7.35 | 0.72 | NA |
|  | *Coronocyclus coronatus* | 5.15 | 1.4 | NA |
|  | *Coronocyclus labratus* | 0.696 | 0.75 | NA |
|  | *Cylicostephanus minutus* | 0 | 21.2 | NA |
|  | *Cyathostomum catinatum* | 0 | 0.995 | NA |
| Oxfendazole + Pyrantel embonate |  |  |  |  |
|  | *Cylicocyclus nassatus* | 29.8 | 56.7 | 11.8 |
|  | *Cylicostephanus longibursatus* | 21.8 | 8.7 | 19 |
|  | *Coronocyclus coronatus* | 16.1 | 13.7 | 64.6 |
|  | *Cylicostephanus minutus* | 8.1 | 14 | 1 |
|  | *Cylicocyclus ashworthi* | 8.01 | 1.4 | 0.4 |
|  | *Cylicocyclus* sp. | 3.12 | 0 | 0.1 |
|  | *Cylicostephanus goldi* | 2.93 | 4.3 | 0.7 |
|  | *Cylicostephanus calicatus* | 2.88 | 0.5 | 0.7 |
|  | *Poteriostomum ratzii* | 2.35 | 0 | 0 |
|  | *Poteriostomum imparidentatum* | 1.08 | 0 | 0 |
|  | *Cyathostomum catinatum* | 1.05 | 0 | 1.6 |
|  | *Cylicocyclus insigne* | 0.747 | 0 | 0 |
|  | *Cyathostomum pateratum* | 0.744 | 0 | 0 |
|  | *Coronocyclus labiatus* | 0.413 | 0 | 0.02 |
|  | *Coronocyclus labratus* | 0.289 | 0.7 | 0.03 |
|  | *Triodontophorus nipponicus* | 0.183 | 0 | 0 |
|  | *Trichostrongylus axei* | 0.108 | 0 | 0 |
|  | *Strongylus vulgaris* | 0.0982 | 0 | 0 |
|  | *Triodontophorus serratus* | 0.0775 | 0 | 0 |
|  | *Cylicostephanus* sp. | 0.0655 | 0 | 0 |
|  | *Triodontophorus brevicauda* | 0.0448 | 0.14 | 0 |
